# Delivery of drinking, eating and mobilising (DrEaMing) and its association with length of hospital stay after major noncardiac surgery: observational cohort study^☆^

**DOI:** 10.1016/j.bja.2022.03.021

**Published:** 2022-05-12

**Authors:** Charles M. Oliver, Samantha Warnakulasuriya, Dermot McGuckin, Georgina Singleton, Peter Martin, Cristel Santos, James Bedford, Duncan Wagstaff, Arun Sahni, David Gilhooly, Jonathan Wilson, Kylie Edwards, Rachel Baumber, Cecilia Vindrola-Padros, Jenny Dorey, Irene Leeman, Hannah Boyd-Carson, Ravi Vohra, Pritam Singh, Matthew Bedford, Abigail Vallance, Giuseppe Aresu, Olga Tucker, Michael Swart, Monty G. Mythen, PQIP project delivery team, Suneetha R. Moonesinghe, for the PQIP collaborative

**Affiliations:** 1 Centre for Perioperative Medicine, Research Department for Targeted Intervention, Division of Surgery and Interventional Science, University College London, London, UK; 2 Department of Anaesthesia and Perioperative Medicine, University College London Hospitals, London, UK; 3 Health Services Research Centre, National Institute for Academic Anaesthesia, Royal College of Anaesthetists, London, UK; 4 Department for Applied Health Research, University College London, London, UK; 5 York University Hospitals, York, UK; 6 Royal National Orthopaedic Hospital, Stanmore, UK; 7 Royal College of Anaesthetists, London, UK; 8 East Midlands Surgical Academic Network, Queen’s Medical Centre, Nottingham, UK; 9 Department of General Surgery, Derby Teaching Hospitals NHS Foundation Trust, Royal Derby Hospital, Derby, UK; 10 Trent Oesophago-Gastric Unit, Nottingham University Hospitals NHS Trust, Nottingham, UK; 11 Regional Oesophago-Gastric Unit, Royal Surrey County Hospital, Guildford, Surrey, UK; 12 Division of Surgery, University Hospitals Birmingham NHS Foundation Trust, Birmingham, UK; 13 Bristol Medical School, University of Bristol, Bristol, UK; 14 Department of Thoracic Surgery, Royal Papworth NHS Foundation Trust, Cambridge, UK; 15 Department of Anaesthesia and Perioperative Medicine, Torbay Hospital, Torquay, UK; 16 University College London Hospitals National Institute of Health Research Biomedical Research Centre, London, UK

**Keywords:** enhanced recovery, patient-centred outcomes, perioperative, quality improvement, quality metric, shared decision-making, surgical outcomes

## Abstract

**Background:**

Enhanced recovery pathways are associated with improved postoperative outcomes. However, as enhanced recovery pathways have become more complex and varied, compliance has reduced. The ‘DrEaMing’ bundle re-prioritises early postoperative delivery of drinking, eating, and mobilising. We investigated relationships between DrEaMing compliance, postoperative hospital length of stay (LOS), and complications in a prospective multicentre major surgical cohort.

**Methods:**

We interrogated the UK Perioperative Quality Improvement Programme dataset. Analyses were conducted in four stages. In an exploratory cohort, we identified independent predictors of DrEaMing. We quantified the association between delivery of DrEaMing (and its component variables) and prolonged LOS in a homogenous colorectal subgroup and assessed generalisability in multispecialty patients. Finally, LOS and complications were compared across hospitals, stratified by DrEaMing compliance.

**Results:**

The exploratory cohort comprised 22 218 records, the colorectal subgroup 7230, and the multispecialty subgroup 5713. DrEaMing compliance was 59% (13 112 patients), 60% (4341 patients), and 60% (3421), respectively, but varied substantially between hospitals. Delivery of DrEaMing predicted reduced odds of prolonged LOS in colorectal (odds ratio 0.51 [0.43–0.59], *P*<0.001) and multispecialty cohorts (odds ratio 0.47 [0.41–0.53], *P*<0.001). At the hospital level, complications were not the primary determinant of LOS after colorectal surgery, but consistent delivery of DrEaMing was associated with significantly shorter LOS.

**Conclusions:**

Delivery of bundled and unbundled DrEaMing was associated with substantial reductions in postoperative LOS, independent of the effects of confounder variables. Consistency of process delivery, and not complications, predicted shorter hospital-level length of stay. DrEaMing may be adopted by perioperative health systems as a quality metric to support improved patient outcomes and reduced hospital length of stay.

**Editor’s key points**

• Enhanced recovery pathways have been widely adopted but implementation can be difficult and high compliance variable.
• A simplified ‘bundle’ approach has been proposed, where the aim is to support patients to DRink free fluids, EAt a soft diet and Mobilise with the maximum assistance of one person (DrEaMing) within 24 h of surgery ending.
• This study evaluated the relationship between DrEaMing and postoperative length of stay in a national cohort of patients undergoing major elective surgery.
• In the overall cohort of 22,218 participants, and in two sub-groups, DrEaMing compliance was around 60% and associated with significantly reduced length of stay, independent of confounders including patient and hospital level factors. DrEaMing was also associated a lower risk of later postoperative complications.
• In a homogenous sub-group of patients undergoing one of four colorectal procedures (n = 7230), the quintile of hospitals with the highest DrEaMing compliance had a 2-day shorter median length of stay than the quintile with the lowest DrEaMing compliance.
• DrEaMing fulfils many of the criteria of an ideal care bundle and improvement target and may be adopted as a quality metric by perioperative health systems.

Enhanced recovery is proposed to reduce the stress response and accelerate recovery from surgery.^1^ The rationale is the prevention of iatrogenic harm from fluid imbalance, avoidance of the deleterious effects of immobility, and promotion of early return of normal homeostasis, by consistently delivering core perioperative processes.^2^ There are limited data indicating cost savings, both to healthcare providers and the wider society,^3^ associated with implementation of enhanced recovery programmes.^4^ Original enhanced recovery pathways (ERPs) comprised four processes, prioritising early mobilisation and resumption of oral diet.^1^ However, ERPs have since become substantially more complex, with between 12 and 20 elements depending on surgery type.^4^ However, although close compliance is associated with improved patient outcomes,^5^ there is widespread evidence that not only the implementation, but also sustained use of ERPs is challenging.^4^ Other key issues include: inadequate or conflicting evidence on the effectiveness of individual processes, resulting in debate over which to mandate and in which subpopulations^6^, ^7^, ^8^, ^9^; and the challenges arising from the shared and often overlapping responsibility for the delivery of ERP processes between multidisciplinary teams across the perioperative period.^10^.^11^

Care bundles are sets of processes which, when delivered together, have been shown to be associated with improved outcomes across a variety of healthcare contexts.^12^ They should comprise no more than three to five component processes, each of which should have robust supporting evidence of efficacy and be supported by a clinical consensus.^13^.^14^ Returning to the core components of ERPs, the ‘DrEaMing’ bundle was developed to promote the delivery of early drinking, eating, and mobilising within 24 h of surgery.^15^ DrEaMing is an attractive process bundle, since both its components and purpose are intuitive, components are not controversial for most procedures, and it fulfils the above requirements for care bundles.^13^ However, few studies have specifically evaluated the impact of DrEaMing on postoperative outcomes, or whether delivery of this bundle improves overall ERP compliance when nested within one.^16^ We hypothesise that, as both a process and outcome, DrEaMing is associated with uncomplicated postoperative recovery and discharge from hospital, and therefore reduced length of postoperative stay. This study therefore interrogates the relationships between DrEaMing and postoperative length of stay (LOS) and complications in a large prospective multispecialty, multicentre cohort of patients undergoing a representative sample of major surgical procedures.

## Methods

The study had three aims: to evaluate the association between delivery of DrEaMing within 24 h of surgery and postoperative LOS; to identify patient and process factors predictive of failure to DrEaM; and to explore the specific confounding effect of postoperative complications on the association between DrEaMing and LOS at hospital level. This manuscript was prepared to comply with STROBE guidelines.^17^

### Data source and approvals

We analysed data from the UK national Perioperative Quality Improvement Programme (PQIP, www.pqip.org.uk) collected for patients who underwent surgery between ^13^ December 2016 and February 28, 2020 (cohort start and end dates determined by the study start date, and the date of the COVID-19 pandemic beginning to impact on hospital processes). PQIP is a prospective observational cohort study of a sample of adults (≥18 yr on date of surgery) undergoing major, planned noncardiac surgery in UK NHS hospitals.^18^, ^19^, ^20^ The full list of eligible operations is available on the PQIP website. Local research teams recruit five eligible patients per specialty at their site each week. Case-mix, process, and outcome data are collected locally and submitted electronically into the web-based study database. PQIP was approved by the Health Research Authority (London–Surrey Research Ethics Committee REC reference number: 16/LO/1827).

### Inclusions

Records were eligible for inclusion if: the patient survived to hospital discharge (Supplementary Fig 1), to exclude misleading short LOS in decedents; data items required to calculate LOS were recorded; LOS exceeded 24 h (to include only major procedures), and no component of DrEaMing was expressly contraindicated based on the surgical procedure performed (Supplementary Table S1). This list of procedures was ratified by expert clinicians. Resulting records comprised an exploratory cohort, within which colorectal and multispecialty subgroups were identified. Colorectal procedures were selected on the grounds of the substantial existing enhanced recovery literature and high volumes of submitted cases to PQIP. Records were eligible for inclusion in the colorectal subgroup if the participant had undergone one of the following four procedures: anterior resection; right hemicolectomy with anastomosis; excision of sigmoid colon; or left hemicolectomy with anastomosis. Records were eligible for inclusion in the multispecialty cohort by surgical procedure, if at least 400 eligible cases were submitted by a minimum of 10 participating hospitals.

### Variable definitions

The primary variable of interest was the composite of drinking, eating, and mobilising (DrEaMing) which is recorded 24 h after surgery by PQIP. DrEaMing is defined by PQIP as drinking (tolerating free fluids within 24 h after completion of surgery), eating (restarted and tolerated at least oral soft diet within 24 h after completion of surgery), and mobilising (mobilised from bed to chair with maximum assistance of one within 24 h after completion of surgery); full definitions are reported in Supplementary Table S2.^21^ Secondary variables of interest were the composite DrEaMing+ (DrEaMing plus the cessation of i.v. fluid administration) within 24 h, and individual DrEaMing+ components. The primary outcome was prolonged postoperative LOS, defined as LOS greater than the 75th centile by individual operative procedure. Secondary outcomes were postoperative LOS (days) and major complications (Clavien–Dindo Grade ≥II).^22^

PQIP categorises operative urgency as expedited (early, where the condition is not an immediate threat to life, limb, or organ survival) or elective (timing to suit patient, hospital, and staff) using the National Confidential Enquiry into Patient Outcome and Death (NCEPOD) classifications.

### Statistical analysis

#### Overview

We first performed exploratory analyses to identify patient, process, and temporal factors independently associated with achievement of DrEaMing at 24 h in the exploratory cohort. We then performed three analyses in the two subgroups. The first analysis evaluated the relationship between delivery of the DrEaMing bundle and prolonged postoperative LOS in colorectal patients, adjusting for case-mix, other processes, complications, and temporal variation (Supplementary Table S3). The second analysis assessed the generalisability of the relationship between bundle delivery and outcome beyond colorectal procedures, in other high-volume major operations. Finally, to examine the association of hospital-level differences in delivery of DrEaMing on LOS, and to further investigate any confounding effect of complications, LOS distributions and incidences of major complications were assessed across quintiles of hospitals, stratified by the proportion of colorectal patients at hospital level who DrEaMed within 24 h of surgery. Only hospitals that submitted five or more eligible colorectal cases were included in the quintile analysis.

#### Modelling approach

Data completeness was assessed in eligible records, and sensitivity analyses performed in ineligible records (Supplementary Table S4). In the exploratory cohort, we identified independent predictors of DrEaMing, including patient, surgical, process, and temporal factors, using single and multilevel multiple logistic regression models. Then, case-mix, surgical, process, and temporal covariates, and major postoperative complications were identified for modelling the associations of interest in colorectal and multispecialty subgroups. Criteria for selection of these covariates were putative association with postoperative LOS, applicability across surgical specialties, and ≥95% completeness in eligible records, regardless of univariate significance (Supplementary Table S3). Specifically, since DrEaMing overlaps enhanced recovery, other enhanced recovery metrics (including carbohydrate loading and postoperative drains) were modelled as confounder variables.^23^

Data distributions were assessed, and univariate analyses performed (χ^2^ or logistic regression) on prolonged LOS. Categorical variables were re-grouped where classes contained few individuals or events, as reported in the results. Continuous data were Winsorised (1st and 99th centiles), centred about their mean and, in the case of non-linear relationship with prolonged LOS, transformed using a closed-test fractional polynomial approach.^24^

A mixed-effects multiple logistic regression model was then constructed on prolonged LOS in the colorectal subgroup, with delivery of DrEaMing at 24 h the fixed effect variable of interest, confounder variables as fixed effects, and random intercepts for hospital identifier codes. Odds ratios (ORs) are reported for fixed effects predictors, and the median OR reported to quantify hospital-level influence on LOS.^25^ The modelling process was repeated in the multispecialty subgroup to assess generalisability beyond the four included colorectal procedures. Two additional models were constructed in the colorectal subgroup to sequentially test the association of DrEaMing+ with prolonged LOS, and component DrEaMing+ processes (drinking, eating, mobilising, and cessation of i.v. fluids) with prolonged LOS.

Analysis and dataset management were performed in Stata®15 (StataCorp LP, College Station, TX, USA).

## Results

The exploratory cohort comprised 22 218 participant records, submitted by 135 hospitals. Of these, 7230 were included in colorectal and 5713 in multispecialty subgroup analyses (Supplementary Fig. S1). We excluded 180 (0.7%) records of participants who died in hospital. Sensitivity analyses indicated lower compliance with DrEaMing, and longer LOS in those who died and those who underwent excluded procedures (Supplementary Table S4). In contrast, compliance with DrEaMing was near-universal in patients discharged within 1 day of surgery. Overall, missingness was extremely low (Supplementary Table S3).

### Exploratory cohort

Median age was 66 yr, 56% were male, and 29% ASA ≥3 (Table 1). Colorectal procedures were most numerous (49.5%) (Table 1). Median postoperative LOS was 6 days (inter-quartile range [IQR] 4–9) overall, but varied by surgical specialty, ranging from 3 days (IQR 2–4) after gynaecological surgery, to 8 days (IQR 5–12) after upper gastrointestinal (GI) and head and neck procedures (Supplementary Table S5).

**Table 1:** Patient characteristics, and perioperative processes and metrics by cohort. Value ranges in parentheses denote inter-quartile ranges and single values percentages.

Factor	Class	Main cohort (*N*=22218)	Colorectal surgery subgroup (*N*=7230)	Multispecialty subgroup (*N*=5713)
*Patient characteristics*				
Age (yr)		66 (56–73)	68 (60–75)	67 (58–73)
Male		12 326 (55.5)	4067 (56.3)	3499 (61.2)
ASA physical status	1	2354 (10.6)	809 (11.2)	531 (9.3)
	2	13 428 (60.4)	4528 (62.6)	3420 (59.9)
	3	6190 (27.9)	1820 (25.2)	1709 (29.9)
	4 or 5	243 (1.1)	72 (1.0)	53 (0.9)
Body mass index (BMI), kg m^−2^		27.1 (24.0–30.8)	27.1 (24.1–30.5)	27.45 (24.39–31.18)
Surgical specialty	Colorectal	11 002 (49.5)	7230 (100.0)	1341 (23.5)
	Hepatobiliary	2015 (9.1)	–	1264 (22.1)
	Orthopaedics	1114 (5.0)	–	427 (7.5)
	Thoracics	1815 (8.2)	–	758 (13.3)
	Urology	2771 (12.5)	–	1923 (33.7)
	Upper GI	837 (3.8)	–	–
	Abdominal - other	708 (3.2)	–	–
	Burns and plastics	460 (2.1)	–	–
	Gynaecology	294 (1.3)	–	–
	Head and neck	543 (2.4)	–	–
	Spinal	496 (2.2)	–	–
	Vascular	163 (0.7)	–	–
Malignancy∗	None	6957 (31.3)	1253 (17.3)	1402 (24.5)
	Primary only	12 015 (54.1)	5118 (70.8)	3281 (57.4)
	Nodal metastases	3246 (14.6)	859 (11.9)	1030 (18.0)
ECG findings∗	Normal	17 232 (77.6)	5589 (77.3)	4419 (77.3)
	AF rate 60–90 beats min^−1^	829 (3.7)	317 (4.4)	205 (3.6)
	AF rate >90 beats min^−1^/other abnormality	2598 (11.7)	902 (12.5)	731 (12.8)
	Not documented	1559 (7.0)	422 (5.8)	4419 (77.3)
Cardiac signs∗	No failure	16 620 (74.8)	5250 (72.6)	4204 (73.6)
	Antihypertensive, diuretic, digoxin	5136 (23.1)	1818 (25.1)	1395 (24.4)
	Peripheral oedema	462 (2.1)	162 (2.2)	114 (2.0)
Heart failure (NYHA) class	I	18 257 (82.2)	5872 (81.2)	4674 (81.8)
	II	3317 (14.9)	1158 (16.0)	903 (15.8)
	III or IV	644 (2.9)	200 (2.8)	136 (2.4)
Dyspnoea∗	None	18 696 (84.1)	6133 (84.8)	4750 (83.1)
	On exertion	2789 (12.6)	879 (12.2)	781 (13.7)
	Limiting exertion/at rest	733 (3.3)	218 (3.0)	182 (3.2)
Respiratory infection in preceding month		781 (3.5)	247 (3.4)	218 (3.8)
Cerebrovascular disease		887 (4.0)	300 (4.1)	240 (4.2)
Dementia		161 (0.7)	48 (0.7)	41 (0.7)
Diabetes mellitus	Non-insulin dependent	2194 (9.9)	766 (10.6)	608 (10.6)
	Insulin-dependent	758 (3.4)	230 (3.2)	219 (3.8)
Liver disease		233 (1.0)	52 (0.7)	87 (1.5)
Smoking History	Never smoked	10 509 (47.3)	3688 (51.0)	2511 (44.0)
	Ex-smoker >6 months	7299 (32.9)	2387 (33.0)	2013 (35.2)
	Ex-smoker <6 months	1124 (5.1)	272 (3.8)	286 (5.0)
	Current smoker	2367 (10.7)	624 (8.6)	641 (11.2)
	Not known	919 (4.1)	259 (3.6)	262 (4.6)
Urgency of surgery	Elective	20 077 (90.4)	6432 (89.0)	5180 (90.7)
	Expedited	2141 (9.6)	798 (11.0)	533 (9.3)
AXA operative severity	Complex/major	14 156 (63.7)	3200 (44.3)	5312 (93.0)
	X-major	8062 (36.3)	4030 (55.7)	401 (7.0)
Operations in preceding month, including index procedure	1	21 322 (96.0)	7018 (97.1)	5441 (95.2)
	2 or more	893 (4.0)	211 (2.9)	272 (4.8)
Planned postoperative level of care	Ward	10 641 (47.9)	4007 (55.4)	2502 (43.8)
	Level 1	2749 (12.4)	1056 (14.6)	622 (10.9)
	Level 2 or enhanced care	7913 (35.6)	2060 (28.5)	2354 (41.2)
	Level 3	915 (4.1)	107 (1.5)	235 (4.1)
*Preoperative physiological and biochemical variables*				
Serum sodium (mmol L^−1^)		140 (138–141)	140 (138–142)	140 (138–141)
Serum potassium (mmol L^−1^)		4.4 (4.1–4.6)	4.4 (4.1–4.6)	4.4 (4.1–4.6)
Serum creatinine (μmol L^−1^)		75 (64–89)	75 (65–88)	77 (66–92)
Serum white cell count (10^9^ L^−1^)		7.2 (5.9–8.8)	7.2 (6–8.7)	7.2 (5.9–8.7)
Serum haemoglobin (g dl^−1^)		13.3 (12.1–14.4)	13.1 (11.7–14.3)	13.5 (12.3–14.6)
HR (beats min^−1^)		76 (67–85)	76 (67–85)	76 (67–85)
Systolic BP (mm Hg)		135 (122–148)	136 (123–148)	136 (124–150)
Peripheral oxygen saturation (%)		98 (96–99)	98 (96–99)	97 (96–98)
*Intraoperative factors*				
Operative approach	Laparoscopic	9892 (44.5)	5372 (74.3)	1710 (30.0)
	Open	10 238 (46.1)	1698 (23.5)	2744 (48.0)
	Robotic	1130 (5.1)	158 (2.2)	792 (13.9)
	Thoracoscopic	958 (4.3)	2 (0.03)	467 (8.2)
Intraoperative blood loss∗	<100 ml	7041 (31.7)	2768 (38.3)	1612 (28.2)
	100–1000	7157 (32.2)	2274 (31.5)	1931 (33.8)
	>1000	2334 (10.5)	312 (4.3)	789 (13.8)
	Recorded missing	5686 (25.6)	1876 (25.9)	1381 (24.2)
Duration of surgery	<2 h	1587 (7.1)	259 (3.6)	290 (5.1)
	2–3 h	5537 (24.9)	1934 (26.7)	1337 (23.4)
	>3 h	15 094 (67.9)	5037 (69.7)	4086 (71.5)
*Perioperative processes and metrics*				
ER protocol used		14 051 (63.2)	5843 (80.8)	3325 (58.2)
Preoperative bowel prep		6634 (29.9)	4312 (59.6)	998 (17.5)
Preoperative carbohydrate loading	Yes	10 765 (48.5)	5216 (72.1)	2417 (42.3)
	Unknown	3221 (14.5)	796 (11.0)	856 (15.0)
Epidural catheter sited		4476 (20.1)	1233 (17.1)	1319 (23.1)
Intrathecal anaesthesia		7595 (34.2)	3724 (51.5)	1673 (29.3)
Regional block used		3078 (13.9)	878 (12.1)	980 (17.2)
Intraoperative depth of anaesthesia monitoring		5997 (27.0)	1840 (25.4)	1498 (26.2)
Intraoperative peripheral nerve stimulator use		7094 (31.9)	2793 (38.6)	1702 (29.8)
Intraoperative temperature probe		16 167 (72.8)	5399 (74.7)	4184 (73.2)
Severity of postoperative pain (in recovery)	None	9206 (41.4)	3190 (44.1)	2369 (41.5)
	Mild	4894 (22.0)	1579 (21.8)	1265 (22.1)
	Moderate	4350 (19.6)	1416 (19.6)	1138 (19.9)
	Severe	2309 (10.4)	657 (9.1)	581 (10.2)
	Unable to assess	1459 (6.6)	388 (5.4)	360 (6.3)
Core temperature >36°C immediately postoperatively		19 716 (88.7)	6399 (88.5)	5043 (88.3)
Abdominal drain(s) sited		9257 (41.7)	2828 (39.1)	2869 (50.2)
Nasogastric tube postoperatively		3252 (14.6)	622 (8.6)	938 (16.4)
Drinking, eating, mobilising, and i.v. fluid cessation within 24 h of surgery				
DrEaM		13 112 (59.0)	4341 (60.0)	3421 (59.9)
DrEaM+		10 348 (46.6)	3367 (46.6)	2650 (46.4)
Drank		19 487 (87.7)	6573 (90.9)	5069 (88.7)
Ate		15 302 (68.9)	4888 (67.6)	4010 (70.2)
Mobilised out of bed		17 207 (77.4)	5925 (82.0)	4349 (76.1)
ntravenous fluids discontinued		13 411 (60.4)	4422 (61.2)	3342 (58.5)

ASA, American Society of Anesthesiologists; AXA, AXA health classification; DrEaM, drinking, eating, and mobilising; GI, gastrointestinal; NYHA, New York Heart Association.

∗ POSSUM (physiological and operative severity score for the enumeration of mortality) classification.

Compliance with DrEaMing was 13 112 (59%) patients within 24 h of surgery overall (Table 1). Compliance was highest for drinking (19 487, [88%] patients) (Table 1). Regression modelling identified several fixed and potentially modifiable factors that predicted DrEaMing at 24 h (Table 2). Advanced functional limitation, and head and neck and upper GI surgery (despite exclusions) predicted failure. Predictors of success included delivery of other ERP components (Table 2), higher preoperative haemoglobin levels, less intraoperative bleeding, and better perioperative pain control; and successive calendar years since 2017. DrEaMing was achieved more often after surgery on Tuesday and Wednesday, compared with the rest of the week, but there was insufficient statistical evidence to be certain about day of the week differences.

**Table 2:** Independent predictors of DrEaMing (drinking, eating, and mobilising) 24 h after surgery in the exploratory cohort, identified using single level and multilevel multivariable logistic regression models. Median OR for multilevel model 0.79 (95% CI 0.68–0.90).

	Single level model		P	Multilevel model		*P*
	OR	95% CI		OR	95% CI	
Case mix descriptors						
Age (yr)	1.00	1.00–1.01	0.10	1.00	1.00–1.00	0.57
Sex						
Male	1.06	0.99–1.14	0.08	1.08	1.01–1.17	0.03
Female	Ref			Ref		
ASA physical status						
1 or 2	Ref			Ref		
3	0.89	0.83–0.97	0.01	0.88	0.81–0.96	0.00
4 or 5	0.82	0.60–1.11	0.19	0.86	0.62–1.18	0.35
Body mass index (BMI), kg m^−2^	1.00	1.00–1.00	0.19	1.00	1.00–1.00	0.37
Serum sodium (mmol L^−1^)	1.01	0.99–1.02	0.34	1.01	1.00–1.02	0.22
Serum potassium (mmol L^−1^)	1.00	1.00–1.00	0.04	1.00	1.00–1.00	0.46
Serum creatinine (μmol L^−1^)∗	1.10	0.91–1.33	0.32	1.15	0.94–1.40	0.17
Serum white cell count (10^9^ L^−1^)	1.00	0.99–1.01	0.69	1.00	0.99–1.01	0.85
Serum haemoglobin (g dl^−1^)	1.03	1.01–1.05	0.00	1.03	1.01–1.05	0.01
Heart rate (beats min^−1^)	1.00	1.00–1.00	0.11	1.00	1.00–1.00	0.10
Systolic pressure (mm Hg)	1.00	1.00–1.00	0.00	1.00	1.00–1.01	0.00
Peripheral O_2_ saturation (%)	0.98	0.96–1.00	0.05	0.99	0.96–1.01	0.25
Malignancy†						
None	Ref			Ref		
Primary only	0.98	0.91–1.07	0.67	1.06	0.97–1.16	0.17
Metastatic	1.11	1.00–1.24	0.06	1.21	1.07–1.36	0.00
Operative urgency						
Elective	Ref					
Expedited	1.03	0.93–1.15	0.58	1.06	0.93–1.20	0.40
ECG abnormalities†						
None	Ref					
AF 60–90	0.98	0.83–1.16	0.83	1.00	0.84–1.20	1.00
AF >90/other	0.96	0.87–1.06	0.45	1.03	0.93–1.15	0.58
Not done	0.98	0.87–1.11	0.80	1.01	0.89–1.16	0.83
Cardiac signs†						
No failure	Ref					
Antihypertensive, diuretic, digoxin	1.01	0.93–1.09	0.82	1.02	0.94–1.11	0.65
Peripheral oedema	1.03	0.83–1.29	0.78	1.04	0.82–1.31	0.77
Dyspnoea (respiratory signs)†						
None	Ref			Ref		
On exertion	0.83	0.75–0.92	0.00	0.86	0.77–0.96	0.01
Limiting exertion	0.91	0.75–1.11	0.35	0.90	0.73–1.10	0.31
NYHA						
I	Ref					Ref
II	0.95	0.86–1.04	0.28	0.88	0.79–0.98	0.02
III or IV	0.76	0.62–0.93	0.01	0.77	0.62–0.96	0.02
Pneumonia in preceding month						
No	Ref					Ref
Yes	0.93	0.78–1.09	0.37	0.91	0.76–1.09	0.30
Cerebrovascular disease						
No	Ref			Ref		
Yes	1.05	0.90–1.22	0.57	1.03	0.87–1.21	0.74
Dementia						
No	Ref			Ref		
Yes	0.81	0.56–1.17	0.26	0.85	0.58–1.24	0.39
Diabetes mellitus						
None	Ref			Ref		
Yes	1.03	0.94–1.14	0.49	0.98	0.88–1.08	0.65
Liver disease						
No	Ref			Ref		
Yes	1.41	1.04–1.90	0.03	1.53	1.11–2.10	0.01
Smoking history						
Never	Ref			Ref		
Quit >6 months previous	0.93	0.87–1.00	0.05	0.95	0.88–1.02	0.18
Current or quit <6 months previous	0.96	0.87–1.05	0.39	0.97	0.88–1.07	0.56
Unknown	1.01	0.86–1.18	0.94	0.99	0.84–1.18	0.92
Temporal factors						
Day surgery was performed						
Sunday	0.84	0.42–1.69	0.62	0.73	0.36–1.47	0.37
Monday	0.91	0.83–1.00	0.05	0.92	0.83–1.01	0.08
Tuesday	Ref			Ref		
Wednesday	1.00	0.91–1.09	0.98	1.00	0.91–1.10	0.96
Thursday	0.90	0.82–0.99	0.02	0.93	0.84–1.03	0.14
Friday	0.86	0.77–0.95	0.01	0.89	0.79–1.00	0.05
Saturday	0.72	0.42–1.23	0.23	0.67	0.38–1.17	0.16
Year index procedure was performed						
2016	1.14	0.17–7.57	0.89	1.16	0.16–8.46	0.88
2017	0.71	0.61–0.82	0.00	0.76	0.64–0.89	0.00
2018	0.82	0.71–0.94	0.01	0.88	0.75–1.02	0.10
2019	0.89	0.78–1.03	0.12	0.92	0.79–1.07	0.29
2020	Ref			Ref		
Perioperative processes and metrics						
Surgical specialty						
Colorectal	Ref			Ref		
Abdominal (other)	1.35	1.12–1.62	0.00	1.24	1.02–1.51	0.03
Burns and plastics	5.70	4.29–7.58	0.00	3.41	2.44–4.77	0.00
Gynaecology	2.96	2.11–4.14	0.00	2.76	1.92–3.97	0.00
Head and neck	1.00	0.79–1.26	1.00	0.59	0.45–0.77	0.00
Hepatobiliary	2.22	1.93–2.55	0.00	2.09	1.75–2.51	0.00
Orthopaedics	1.27	1.06–1.52	0.01	1.83	1.44–2.33	0.00
Spinal	1.90	1.51–2.40	0.00	2.77	2.02–3.80	0.00
Thoracics	5.10	4.08–6.37	0.00	6.18	4.80–7.95	0.00
Upper GI	0.56	0.46–0.68	0.00	0.52	0.42–0.65	0.00
Urology	2.25	1.99–2.53	0.00	2.23	1.96–2.55	0.00
Vascular	2.14	1.47–3.11	0.00	1.54	1.04–2.29	0.03
Operative approach						
Open	Ref.			Ref		
Laparoscopic	1.44	1.34–1.56	0.00	1.45	1.34–1.58	0.00
Robotic	1.02	0.87–1.20	0.78	1.18	0.99–1.41	0.07
Number of preceding operations during index admission†						
None	Ref.			Ref		
≥1	0.80	0.68–0.94	0.01	0.87	0.73–1.03	0.10
AXA operative severity code						
Complex/major	Ref.			Ref		
X-major	0.95	0.88–1.02	0.17	0.98	0.91–1.06	0.60
Enhanced recovery protocol used						
No	Ref.			Ref		
Yes	1.31	1.22–1.41	0.00	1.18	1.09–1.29	0.00
Preoperative bowel preparation administered						
No	Ref.			Ref		
Yes	0.96	0.89–1.04	0.31	0.92	0.85–1.01	0.08
Preoperative carbohydrate loading						
No	Ref.			Ref		
Yes	1.15	1.06–1.25	0.00	0.92	0.84–1.01	0.09
Unknown	0.99	0.89–1.09	0.78	0.86	0.77–0.96	0.01
Perioperative epidural analgesia						
No	Ref.			Ref		
Yes	0.82	0.75–0.89	0.00	0.67	0.61–0.75	0.00
Intrathecal anaesthesia/analgesia						
No	Ref.			Ref		
Yes	1.08	1.00–1.16	0.04	1.06	0.97–1.15	0.22
Regional anaesthesia/analgesia						
No	Ref.			Ref		
Yes	1.00	0.91–1.11	0.97	0.94	0.84–1.04	0.24
Intraoperative depth of anaesthesia monitoring						
No	Ref.			Ref		
Yes	0.92	0.85–0.99	0.02	1.02	0.94–1.12	0.60
Intraoperative neuromuscular monitoring						
No	Ref.			Ref		
Yes	1.03	0.96–1.10	0.48	1.06	0.98–1.15	0.13
Intraoperative temperature probe used						
No	Ref.			Ref		
Yes	1.08	0.98–1.19	0.11	1.03	0.93–1.15	0.55
Duration (h)						
<2	Ref.			Ref		
2-3	0.72	0.62–0.84	0.00	0.73	0.62–0.85	0.00
>3	0.51	0.44–0.60	0.00	0.52	0.44–0.61	0.00
Blood loss (ml)						
<500	Ref.			Ref		
500–1000	0.77	0.71–0.83	0.00	0.77	0.71–0.85	0.00
>1000	0.54	0.48–0.61	0.00	0.56	0.49–0.63	0.00
Missing data	0.80	0.73–0.87	0.00	0.77	0.70–0.85	0.00
Postoperative level of care						
Ward	Ref.			Ref		
1	0.66	0.60–0.73	0.00	0.70	0.62–0.79	0.00
1.5 or 2	0.50	0.47–0.54	0.00	0.48	0.44–0.53	0.00
3	0.31	0.26–0.37	0.00	0.26	0.21–0.31	0.00
Severity of immediate postoperative pain						
None	Ref.			Ref		
Mild	0.95	0.88–1.03	0.23	0.95	0.87–1.03	0.22
Moderate	0.89	0.82–0.97	0.01	0.90	0.82–0.99	0.03
Severe	0.69	0.62–0.77	0.00	0.72	0.64–0.80	0.00
Unable to assess	0.60	0.52–0.69	0.00	0.70	0.60–0.81	0.00
Core temperature >36°C in recovery room						
No	Ref.			Ref		
Yes	0.90	0.84–0.97	0.01	0.97	0.89–1.05	0.44
Intra-abdominal drain in place postoperatively						
No	Ref.			Ref		
Yes	0.66	0.62–0.71	0.00	0.70	0.64–0.75	0.00
Nasogastric tube in place postoperatively						
No	Ref.			Ref		
Yes	0.33	0.29–0.36	0.00	0.32	0.29–0.36	0.00

ASA, American Society of Anesthesiologists; AXA, AXA health classification; CI, confidence interval; GI, gastrointestinal; NYHA, New York Heart Association; OR, odds ratio.

∗ Quadratic transformation.

† POSSUM (physiological and operative severity score for the enumeration of mortality) classification.

Without adjustment for confounders, DrEaMing patients had a 3 day shorter median LOS {5 days (95% confidence interval [CI] 5–5 days)} than those who did not DrEaM (8 days [95% CI 8–8 days]). DrEaMing+, and each individual component, were also associated with shorter median LOS, with 95% CIs indicating reductions of between 2 and 5 days (Supplementary Table S6).

Overall, 5638 patients (25.4%) developed at least one major postoperative complication during their admission, but this varied by delivery of DrEaMing: the incidence of one or more major complications was 37% among patients who failed to DrEaM and 17% in those who achieved DrEaMing (Supplementary Table S7). Major pulmonary (3.7 *vs* 1.9), cardiovascular (4.8 *vs* 1.9), and GI (20.7 *vs* 6.3) complications were more common in those who failed to DrEaM than in those who DrEaMed (Supplementary Table S7).

### Colorectal subgroup

Eligible records were submitted by 113 hospitals, with a median of 55 records per hospital. Patient characteristics were generally similar to the main cohort (Table 1), with a few notable exceptions: colorectal surgery tended to be more complex (56% complex major, in comparison with 36%), was more frequently for cancer resection (83% *vs* 69%), and enhanced recovery protocols were used with greater consistency (80% *vs* 63%) overall.

In total 4341 (60%) patients DrEaMed within 24 h of surgery (Table 1) but the degree of variation between hospitals was substantial (median 63%, IQR 37–73%, range 0–100%; Supplementary Fig. S2). As with the main exploratory cohort, compliance was highest with drinking (6573 [91%] patients; Table 1).

Median postoperative LOS in the colorectal subgroup was 6 (IQR 4–9) days overall, with similar distributions by individual operative procedure (Supplementary Table S8). Prolonged LOS was therefore defined as postoperative LOS exceeding 8 days after right or left hemicolectomy with anastomosis, or exceeding 10 days after anterior resection or sigmoid colectomy. LOS varied markedly between hospitals (Supplementary Fig. S3). Delivery of DrEaMing was associated with a 2-day shorter median LOS (5 days [95% CI 5–5 days]), compared with failure (7 days [95% CI 7–7 days]) (Supplementary Table S9). DrEaMing+, and each individual component, were also associated with shorter median LOS, with 95% CIs indicating reductions of between 2 and 3 days (Supplementary Table S9). Major postoperative complications were more common in patients who did not DrEaM at 24 h (32%) compared with those who did (17%) (Supplementary Table S7).

In multilevel analysis, delivery of DrEaMing was associated with substantially reduced odds of prolonged LOS (OR 0.51 [95% CI 0.43–0.59]), controlling for measured confounders (Table 3). Median odds ratio (MOR) was 0.56 (0.45–0.71), indicating substantial influence of unmeasured hospital-level effects on the outcome. DrEaMing+ was associated with similar magnitude odds reduction (OR 0.54 [0.46–0.63]), and the component variables with ORs of between 0.66 and 0.77 (Table 4).

**Table 3:** Predictors of prolonged postoperative length of stay after colorectal surgery: multilevel analysis testing DrEaMing (drinking, eating, and mobilising) as the variable of interest (median odds ratio 0.56 [0.45–0.71] P<0.001). AF, atrial fibrillation; CI, confidence interval.

Variable	Odds ratio	95% CI	*P*
Variable of interest			
DrEaMing status at 24 h			
Delivered	0.51	0.43–0.59	0.00
Not delivered	Ref		
Case-mix variables			
Preoperative physiological and biochemical variables			
Age (yr)	1.02	1.01–1.03	0.00
Body mass index (BMI), kg m^−2^	1.01	1.00–1.01	0.05
Serum [sodium]	0.99	0.97–1.02	0.52
Serum [potassium]	0.96	0.80–1.14	0.71
Serum [creatinine]	0.64	0.40–1.02	0.06
Serum white cell count	1.03	0.99–1.06	0.10
Serum [haemoglobin]	0.99	0.95–1.04	0.69
HR	1.00	1.00–1.01	0.15
Systolic BP	1.00	1.00–1.00	0.97
Oxygen saturations	0.96	0.92–1.01	0.14
Sex			
Male	1.38	1.17–1.63	0.00
Female	Ref		
ASA physical status			
1	Ref		
2	0.81	0.62–1.04	0.10
3	1.10	0.82–1.49	0.52
4 or 5	1.56	0.79–3.07	0.20
Malignancy∗			
No solid tumour	Ref		
Local disease	0.99	0.80–1.22	0.92
Nodal or metastatic spread	1.03	0.78–1.36	0.82
Preoperative ECG∗			
No abnormality	Ref		
AF: rate 60–90 beats min^−1^	1.00	0.72–1.40	0.99
AF >90/other abnormality	0.90	0.72–1.11	0.32
ECG was not performed	1.13	0.83–1.54	0.44
Cardiac findings∗			
No failure	Ref		
Diuretic/antihypertensive	1.09	0.91–1.30	0.33
Peripheral oedema/cardiomegaly	1.14	0.72–1.80	0.58
Dyspnoea∗			
None			
On exertion	0.95	0.76–1.19	0.65
Limiting exertion/at rest	0.92	0.61–1.39	0.70
NYHA class			
I	Ref		
II	1.11	0.89–1.37	0.35
III or IV	0.98	0.63–1.52	0.93
Cerebrovascular disease			
History	1.32	0.95–1.82	0.09
No history	Ref		
Dementia			
History	1.66	0.76–3.60	0.20
No history	Ref		
Diabetes mellitus			
None	Ref		
Non-insulin-dependent	1.11	0.89–1.39	0.34
Insulin-dependent	0.92	0.62–1.35	0.66
Liver disease			
History	1.89	0.93–3.83	0.08
No history	Ref		
Smoking history			
Never smoked	Ref		
Unknown	1.18	0.80–1.76	0.40
Quit >6 months ago	1.08	0.92–1.26	0.37
Quit <6 months ago	1.18	0.81–1.72	0.39
Current smoker	1.21	0.93–1.58	0.16
Operative urgency			
Elective	Ref		
Expedited	0.89	0.69–1.14	0.35
Major postoperative complication(s)			
≥1	12.59	10.78–14.69	0.00
None	Ref		
Perioperative processes and metrics			
Operation			
Anterior resection	Ref		
Right hemicolectomy	1.09	0.90–1.33	0.36
Sigmoid colectomy	0.89	0.68–1.16	0.38
Left hemicolectomy	1.28	0.92–1.79	0.15
Number of preceding operations during this admission			
None	Ref		
≥1	1.23	0.82–1.84	0.33
Mode of surgery			
Open	Ref		
Laparoscopic/robotic	0.59	0.50–0.69	0.00
Enhanced recovery protocol			
Used	1.04	0.84–1.28	0.73
Not used	Ref		
Preoperative bowel preparation administered			
Yes	0.95	0.80–1.13	0.55
None	Ref		
Preoperative carbohydrate administered			
Yes	0.98	0.79–1.21	0.84
Unknown	1.23	0.92–1.63	0.16
None	Ref		
Perioperative epidural analgesia			
Yes	1.45	1.16–1.82	0.00
None	Ref		
Intrathecal analgesia			
Yes	0.96	0.81–1.14	0.64
None	Ref		
Regional anaesthesia/analgesia			
Yes	0.94	0.74–1.18	0.60
None	Ref		
Intraoperative depth of anaesthesia monitoring			
Used	0.93	0.77–1.12	0.46
Not used	Ref		
Intraoperative neuromuscular monitoring			
Used	0.93	0.79–1.09	0.35
Not used	Ref		
Intraoperative temperature probe use			
Used	0.93	0.77–1.11	0.40
Not used	Ref		
Duration of surgery			
<2 hours	Ref		
2–3 hours	0.78	0.51–1.18	0.24
>3 hours	0.98	0.65–1.49	0.94
Postoperative level of care			
0	Ref		
1	1.09	0.84–1.42	0.50
1.5 or 2	1.24	1.02–1.50	0.03
3	1.55	0.99–2.43	0.06
Severity of postoperative pain in recovery			
None	Ref		
Mild	1.15	0.95–1.39	0.14
Moderate	1.05	0.86–1.28	0.66
Severe	1.36	1.05–1.77	0.02
Unable to assess	1.03	0.74–1.44	0.84
Core temperature >36°C in recovery room			
Yes	1.19	0.94–1.50	0.16
No	Ref		
Intra-abdominal drain in place postoperatively			
Yes	1.43	1.21–1.70	0.00
None	Ref		
Nasogastric tube in place postoperatively			
Yes	1.63	1.27–2.09	0.00
None	Ref		
Day of the week surgery was performed			
Monday	1.05	0.84–1.31	0.66
Tuesday	Ref		
Wednesday	1.01	0.82–1.25	0.91
Thursday	1.03	0.83–1.27	0.82
Friday	0.86	0.67–1.11	0.26
Saturday	1.12	0.24–5.33	0.89
Sunday	1.24	0.14–11.35	0.85

∗ POSSUM (physiological and operative severity score for the enumeration of mortality) or combined POSSUM categories.

**Table 4:** Independent predictors of prolonged postoperative length of stay—multilevel analysis in the colorectal cohort testing DrEaMing+ (DrEaMing plus the cessation of i.v. fluid administration) component variables (median odds ratio 0.45 [0.35–0.56]). Covariates were modelled to adjust for confounding, but their estimates are not reported here. CI, confidence interval; DrEaMing, drinking, eating, and mobilising.

Processes delivered 24 h after surgery	Odds ratio	95% CI	*P*
Drinking	0.66	0.52–0.84	<0.001
Not drinking	Ref.		
Eaten	0.72	0.54–0.97	0.03
Not eaten	Ref.		
Mobilised	0.66	0.53–0.83	<0.001
Not mobilised	Ref.		
I.V. fluids discontinued	0.77	0.66–0.91	<0.001
I.V. fluids not discontinued	Ref.		

**Fig 1:**
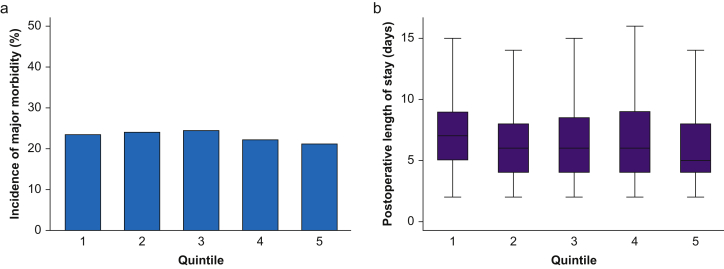
Quintile analysis, in which hospitals were stratified on the distribution of the proportion of patients DrEaMing (drinking, eating, and mobilising) per hospital (q20: 33%, q40: 45%, q60: 67%, q80: 81%). (a) Incidence of major postoperative morbidity (Clavien-Dindo ≥Grade II), range 21–24% at Q5 and Q3 hospitals, respectively (P=0.21). (b) Postoperative median length of stay, 7 (5–9) days at Q1 hospitals and 5 (4–8) days at Q5 hospitals (P<0.001, Kruskall–Wallis).

Stratified by DrEaMing delivery, the highest performing quintile of hospitals delivered DrEaMing in >80% of colorectal surgical patients, contrasting with delivery to fewer than 33% in the lowest performing hospitals. Postoperative LOS was significantly shorter at the highest quintile hospitals (median 5 days [95% CI 5–6 days]) than the lowest (median 7 days [95% CI 6–7 days]), Kruskall–Wallis *P*<0.001 (Fig 1). Although a ‘dose response’ was not evident across the quintiles, these observations suggest that consistent process delivery was associated with shorter LOS. Contrasting with variation in LOS, the incidence of major postoperative complications varied by only 3% between quintiles, supporting our multilevel findings of an association between DrEaMing and shorter LOS, and indicating that complications were not the primary determinant of LOS (Fig 1).

### Multispecialty subgroup

This subgroup comprised 5713 patients undergoing thoracic, urological, hepatobiliary, orthopaedic, and colorectal operations (Supplementary Table S8). Case volume ranged from 758 (thoracoscopic lobectomy) to 401 (right hemicolectomy with ileostomy). In contrast with the colorectal cohort, patients were more comorbid (31% ASA physical status >2) but underwent proportionately less invasive, open surgery. Median postoperative LOS ranged from 2 days after radical prostatectomy to 10 days after Whipple's procedure, with prolonged LOS ranging from >4 days to >15 days, respectively (Supplementary Table S9). Whereas these patients were less frequently enrolled onto ERPs than colorectal cohort patients, the proportion of DrEaMing patients was identical between subgroups (Table 1). Repeating the process of multilevel multiple logistic regression on prolonged postoperative LOS in this cohort yielded a similar odds reduction associated with DrEaMing 24 h after surgery to that observed in the colorectal cohort (OR 0.53 [95% CI: 0.45–0.63], *P*<0.001; Supplementary Table S10).

## Discussion

This study is the first to evaluate the association between DrEaMing compliance and postoperative LOS after major surgery in a large multicentre cohort. We report five key findings. First, delivery of DrEaMing and its component variables were each associated with an approximately halved likelihood of prolonged LOS, both after colorectal surgery and after a representative selection of thoracic, orthopaedic, hepatobiliary, and urological procedures. Second, of the case-mix and process factors that predicted whether patients achieved DrEaMing, all except surgical specialty are potentially modifiable. Third, only the development of a major postoperative complication carried a greater risk of prolonged LOS than failure to deliver DrEaMing. Fourth, at hospital level, major complications was not the primary determinant of LOS after colorectal surgery, instead consistent delivery of DrEaMing was associated with significantly shorter LOS. Finally, we note that epidural catheters, nasogastric tubes, abdominal drains, and experiencing severe pain immediately after surgery were each independently associated both with failure to DrEaM and prolonged LOS.

For improvement endeavours to be effective, it is important to understand the level of compliance with processes of interest, and the barriers to and facilitators of this compliance. The challenges of implementation and maintenance of and adherence to ERPs are well documented, despite national programmes to support their delivery.^4^.^26^ Previous analysis of the PQIP cohort by our group has found consistently high compliance for some enhanced recovery elements, including preoperative assessment, perioperative antibiotic prophylaxis, and temperature management.^27^ Monitoring, rather than high-intensity improvement endeavours, is appropriate for this group of ‘normalised’ processes. Technical interventions, most notably minimal access approaches, and avoidance of nasogastric tubes and abdominal drains, have proved more resistant to change at hospital level.^27^.^28^ These types of process require detailed investigation of local case mix, infrastructure, attitudes, and behaviours in order to understand variation and challenge dogmatism, where appropriate. The evidence for processes such as goal-directed fluid optimisation, bowel preparation, and carbohydrate loading remains contested.^7^.^29^, ^30^, ^31^

It could be argued that system change should not be attempted until evidence from a randomised trial is available, but in contrast with the processes discussed above, the DrEaMing bundle is an ideal target for locally driven quality improvement: its rationale is simple (supporting its inclusion in a care bundle); there is variation in compliance rates between hospitals; but also evidence that improvement, to achieve consistent delivery of the bundle (to at least 80% of patients), is achievable.^14^ Furthermore, since ER programmes are associated with cost savings for healthcare providers,^4^ streamlining complex ERPs to re-prioritising DrEaMing may deliver financial incentives. In recognition of its clinical, patient-centric, and organisational importance, subsequent to our analyses, NHS England has prioritised DrEaMing as a core clinical priority area (commissioning for quality and innovation, CQUIN), in which improvement is expected in 2022/23.^32^ Targeted initiatives to support DrEaMing implementation will need to reach beyond the clinical domain, to involve organisational culture (specifically staff attitudes and behaviours), and structural factors (including finances and staffing), since these have been shown to influence the success of change programmes.^11^^,^^33^

Whether DrEaMing is a process or an outcome is a matter for debate. Our assertion is that it is both. DrEaMing provides a useful target around which further process evaluation can be based—for example, ensuring that all of the required actions to support drinking within 24 h have been delivered—such as clear instructions from the surgical/perioperative team to the ward team, provision of oral fluids, adequate analgesia and antiemesis, and so on. To that end, we can map the processes required to achieve each element of DrEaMing and hypothesise about reasons for success or failure, which would explain between-hospital (rather than between-patient) variation. For example, our weak signal (wide CIs around point estimates) that DrEaMing may be less frequently delivered over weekends, has face validity because processes such as mobilisation may be impeded by workforce constraints (e.g. access to physiotherapists). Opportunities for improvement can therefore also be developed—for example, alternative approaches such as nurse- or therapy assistant-led interventions may provide solutions to workforce challenges.^34^ In hospitals with reliable processes of care and a culture which promotes DrEaMing, failure to achieve the process/early outcome of DrEaMing may highlight patients who require more attention from healthcare staff, as they are at higher risk of later complications and extended LOS. Although we do not assume a causal relationship between failure to DrEaM and postoperative complications, if evaluating against the Bradford Hill criteria,^35^ there is a strong case: strength of association; consistency of findings (if considering previous data from ERPs); temporal sequence; coherence; and biological plausibility (through reduction of fluid imbalance, nutritional depletion, and the complications of immobility, including respiratory impairment).

Strengths of this study include the systematic interrogation of a perioperative dataset of case-mix, process, and outcome variables, unparalleled in its comprehensiveness. Very few records were excluded as a result of missing data items. We expect our findings to be generalisable across healthcare systems because of the diversity of the population represented by participating hospitals, the clear definitions and low complexity of the DrEaMing intervention. There are, however, limitations to these analyses: causes of failure to DrEaM, most notably where driven by local protocols, were not available for interrogation; we did not investigate causality, so, for example, the organisational infrastructure and culture that drives high compliance with the DrEaMing bundle might also be responsible for more efficient discharge from hospital after surgery; some potential confounders (including frailty and operative blood loss) were not modelled because of missingness in or absence from the dataset; findings may not be generalisable beyond the procedures and specialties assessed; and because this was a study of UK patients treated in the NHS, findings may not be generalisable to populations with lower living standards and without comprehensive healthcare coverage. Exclusion of the small number of decedents from our analysis is unlikely to have biased our findings, as a result of the low incidence of DrEaMing in these individuals and the strength of the association between major complications and prolonged LOS. We were unable to investigate associations between shorter postoperative LOS and post-discharge outcomes, and future analyses incorporating readmission, discharge destination, and mortality data are merited. Finally, important questions have been raised that this study was not designed to answer. These include identifying which patients do benefit from perioperative epidural anaesthesia, developing better approaches to postoperative analgesia, and evaluating the role of nasogastric tubes (NGTs) and abdominal drains in contemporary practice.

In summary, in this observational study, delivery of DrEaMing was associated with a 3 day reduction in LOS after a wide variety of major surgical procedures. The direction of this association was independent of confounders, including complications, with DrEaMing associated with a 41–57% reduction in the odds of prolonged hospital stay. Consistency of DrEaMing rather than incidence of complications predicted hospital-level LOS. Taken together, our analyses indicate that substantial gains may be realised, both for patients and organisations, from targeting the consistent delivery of DrEaMing. Furthermore, where early drinking, eating, mobilising, or early cessation of i.v. fluids are individually contraindicated, delivery of remaining processes may be beneficial. DrEaMing appears, therefore, to be a valuable quality metric and a DrEaMing bundle may be an important intervention in enhanced recovery programmes. An RCT would, however, be required to establish causation.

## Authors' contributions

Study conception: SRM, MGM

Study design; data analysis and interpretation; manuscript first draft and revisions: CMO

Study design, manuscript first draft, and revisions: SW

Study design, manuscript revisions: SRM, DM and GS

Statistical overview and revisions: PM

Study conduct and manuscript revisions: PM, JB, DW, AS, JW, KE, RB, CVP, JD, IL, HBC, RV, PS, MB, AV, GA, OT, MS, MGM.

## Funding

The Perioperative Quality Improvement Programme (PQIP) is funded by the 10.13039/501100001297Royal College of Anaesthetists, UK; the 10.13039/501100000765University College London/University College London Hospitals Surgical Outcomes Research Centre, UK; and by the 10.13039/501100000724Health Foundation, UK. NIAA RCoA/BJA project grant to DMG. University College London/University College London Hospitals National Institute for Health Research (NIHR) Biomedical Research Centre to SRM and MGM. All views expressed here are those of the authors and not of the NIHR or Department of Health and Social Care.

## Data availability

The data used in this study are available upon request and after approval from the Perioperative Quality Improvement Programme (PQIP) Project Team at the Royal College of Anaesthetists (https://pqip.org.uk/pages/datarequest).

## Declaration of interest

All authors declare: no support from any organisation for the submitted work; no financial relationships with any organisations that might have an interest in the submitted work in the previous three years; no other relationships or activities that could appear to have influenced the submitted work.
