# Supplementary material for: Delivery of drinking, eating and mobilising (DrEaMing) and its association with length of hospital stay after major noncardiac surgery: observational cohort study
Source: Br J Anaesth. 2022 May 12;129(1):114–26. doi: 10.1016/j.bja.2022.03.021 (PMC9284668; doi:10.1016/j.bja.2022.03.021)
Supplement: Multimedia component 1 [file mmc1.docx]

# Supplementary figures and tables

Table 1 Procedures excluded from exploratory analysis of DrEaMing

| Surgical subtype | Procedure |
| --- | --- |
| Upper GI | Laparotomy + restoration of intestinal continuity |
| Upper GI | Complex restoration of intestinal continuity |
| Upper GI | Partial gastrectomy (+/- excision of surrounding tissue) |
| Upper GI | Open excision of lesion of oesophagus |
| Upper GI | Transthoracic repair of diaphragmatic hernia (acquired) |
| Upper GI | Transthoracic repair of para-oesophageal hiatus hernia |
| Upper GI | Anastomosis of gall bladder (to another viscus) |
| Upper GI | Anastomosis of hepatic duct |
| Upper GI | Anastomosis of pancreatic duct (to another viscus) |
| Upper GI | Repair of bile duct |
| Upper GI | Laparoscopic biliary gastric bypass |
| Upper GI | Revision of gastro-jejunostomy |
| Upper GI | Total or Partial gastrectomy and excision of surrounding tissue |
| Upper GI | Transabdominal repair of diaphragmatic hernia |
| Upper GI | Transthoracic fundoplication |
| Colorectal | Laparotomy for enterocutaneous fistula |
| Hepatobiliary | Frey's procedure |
| Hepatobiliary | Partial excision of bile duct and anastomosis of bile duct to duodenum/jejunum |
| Hepatobiliary | Anastomosis of gall bladder (to another viscus) |
| Hepatobiliary | Anastomosis of hepatic duct |
| Hepatobiliary | Anastomosis of pancreatic duct (to another viscus) |
| Hepatobiliary | Repair of bile duct |
| Hepatobiliary | Total pancreatectomy |
| Hepatobiliary | Pancreatoduodenectomy, excision of surrounding tissue (Whipple's procedure) |
| Head and neck | Partial pharyngectomy |
| Head and neck | Total pharyngectomy |
| Head and neck | Craniofacial resection |
| Head and neck | Partial or Hemi-maxillectomy for malignancy |
| Head and neck | Vertical hemi-laryngectomy |
| Head and neck | Horizontal supra-glottic laryngectomy |
| Head and neck | Reconstruction of larynx with graft |
| Head and neck | Tongue flap - first stage and second stage |
| Head and neck | Extensive excision of mandible ( +/- disarticulation / reconstruction) |
| Head and neck | Total glossectomy |
| Head and neck | Partial laryngectomy |
| Head and neck | Sub-total laryngectomy |
| Head and neck | Total laryngectomy |
| Abdominal Other | Laparotomy + excision of sarcoma tumour |
| Abdominal Other | Total exenteration of pelvis |
| Orthopaedics | Complex pelvic osteotomies and fixation, e.g. triple osteotomy, peri-acetabular osteotomy |
| Orthopaedics | Hemipelvectomy |
| Orthopaedics | Sacrectomy |
| Orthopaedics | Radical clearance of sarcoma of trunk or limbs, +/- amputation or insertion of prosthesis |
| Orthopaedics | Forequarter amputation |
| Orthopaedics | Hindquarter amputation |
| Spinal | Revisional anterior discectomy (cervical region) |
| Spinal | Trans-oral surgery including posterior fixation |
| Spinal | Anterior discectomy (cervical region) |
| Spinal | Combined anterior and posterior fusion of cervical spine |

Table 2 PQIP variable definitions. From standard operating procedures document

| **Postoperative visit, on day 2 or day 3** | | |
| --- | --- | --- |
| Answer these questions with regard to the patient’s status on post-operative day 1 (within 24 hours from completion of surgery). These assess achievement of the enhanced recovery objectives of the CHEERS-DREAM campaign. | | |
| Maintenance IV fluids discontinued within 24hr of surgery ending | Y/N | Select yes if IV maintenance fluids were discontinued within 24 hours of surgery ending. |
| Started drinking (free fluids) within 24hr of surgery ending | Y/N | Select yes if the patient was tolerating free fluids within 24 hours after completion of surgery. No minimum volume currently specified. See web tool for links to recent guidelines. |
| Started eating (at least soft diet) within 24hr of surgery ending | Y/N | Select yes if the patient restarted oral diet (at least soft diet e.g. yoghurt, porridge, fruit) within 24 hours of completion of surgery and tolerated it. This does not have to be full normal diet. If no, indicate if the patient received supplementary nutrition within 24hr of surgery ending. If they did receive supplementary nutrition, indicate the type. |
| Mobilising from bed to chair with max assistance of one person within 24hr of surgery ending | Y/N | Select yes if the patient mobilised from bed to chair with maximum assistance of one person within 24 hours of completion of surgery. |

Table 3 Candidate covariates for multivariable modelling. *: POSSUM classification, ^#^: Enhanced recovery protocol elements, ^$^: excluded from models due to missingness >5%

| Casemix factors | Perioperative processes and metrics |
| --- | --- |
| Age | Operative procedure |
| Sex | Operative severity |
| ASA-PS | Number of preceding operations this admission* |
| BMI^$^ | Incision type^#^ |
| Serum sodium | ERAS protocol used |
| Serum potassium | Preoperative bowel preparation (avoidance of)^#^ |
| Serum urea^$^ | Preoperative carbohydrate loading^#^ |
| Serum creatinine | Perioperative epidural analgesia/ anaesthesia^#^ |
| Serum white cell count | Intrathecal anaesthesia/analgesia^#^ |
| Haemoglobin concentration | Regional anaesthesia^#^ |
| Heart rate | Intraoperative blood loss*^, $^ |
| Systolic blood pressure | Intraoperative soiling (finding)* |
| Oxygen saturations | Intraoperative depth of anaesthesia monitoring |
| Glasgow coma scale | Intraoperative neuromuscular monitoring |
| Malignancy* | Intraoperative temperature monitoring |
| ECG findings* | Duration of surgery |
| Cardiac findings* | Postoperative level of care |
| Respiratory findings* | Postoperative pain severity |
| Frailty^$^ | Postoperative normothermia achieved |
| NYHA class | Postoperative abdominal drain (avoidance of) ^#^ |
| Pneumonia in preceding month | Postoperative nasogastric tube (avoidance of)^#^ |
| Cerebrovascular disease | Temporal |
| Dementia | Year |
| Diabetes mellitus | Day of week of surgery |
| Liver disease | Postoperative complications |
| Smoking history | Clavien-Dindo Grade ≥II |
| Operative urgency |  |

Table 4 Sensitivity analyses

|  | Exclusion criteria | Patients DrEaMing 24-hours after surgery (%) | Postoperative LOS (median (IQR)), days |
| --- | --- | --- | --- |
| Included n=22,218 | - | 13,112 (59.0) | 6 (4-9) |
|  |  |  |  |
| Excluded n=3,305 | All | 2,120 (64.2) | 1 (1-8) |
|  | Died in hospital  n=180 | 45 (25.0) | 11.5 (5-25) |
|  | Incomplete LOS items  n=211 | 58 (27.5) | - |
|  | LOS ≤1 day  n=1833 | 1762 (96.1) | 1 (1-1) |
|  | Surgery after 28 February 2020 n=478 | 343 (71.8) | 4 (2-7) |
|  | Excluded procedures  n=1,941 | 585 (30.1) | 9 (6-15) |

Figure 1 Flow diagram


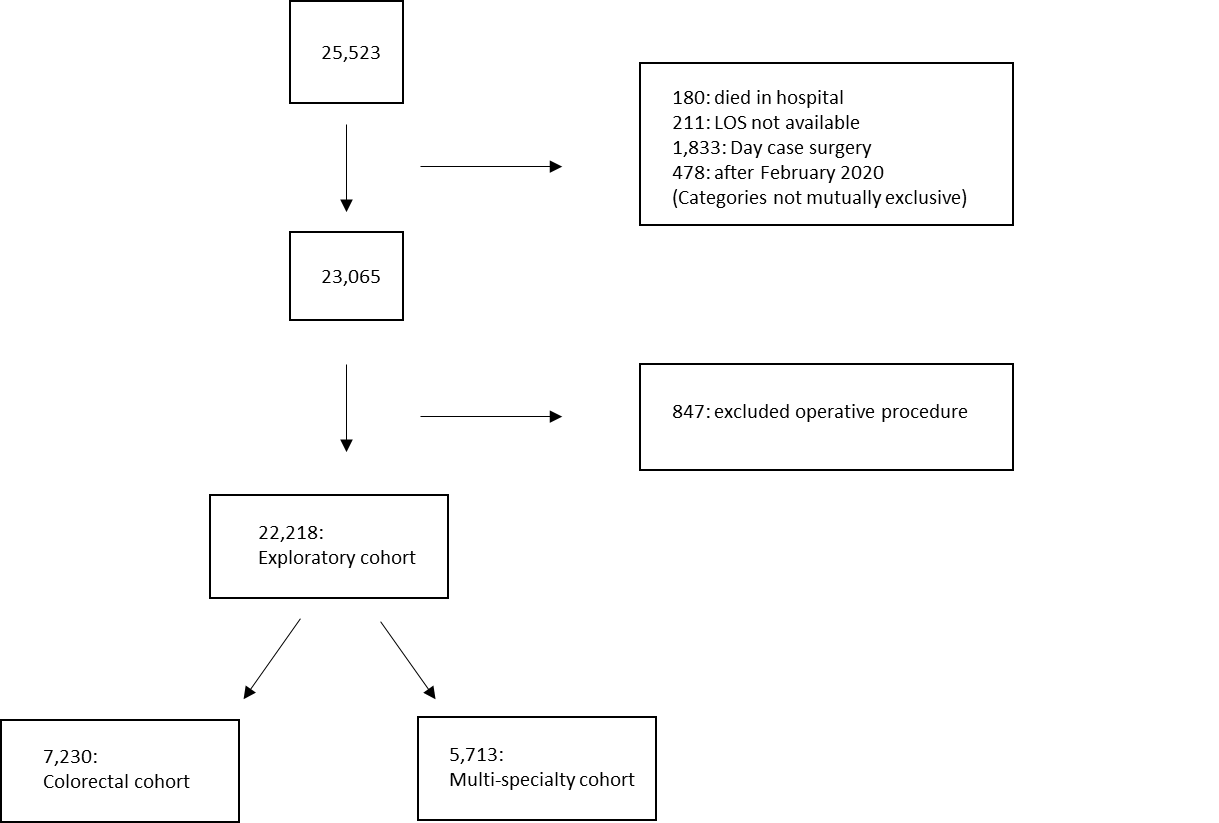


Table 5 Postoperative length of stay by surgical specialty in the exploratory cohort

| Surgical Specialty | N | Median, 95%CI  (days) | IQR (days) | Range (days) |
| --- | --- | --- | --- | --- |
| Hepatobiliary | 2015 | 7, 7-7 | 5 – 11 | 2 - 156 |
| Colorectal | 11002 | 6, 6-6 | 4 – 9 | 2 - 228 |
| Upper GI | 837 | 8, 7-8 | 5 – 12 | 2 - 107 |
| Abdominal - other | 708 | 7, 6-7 | 4 – 12 | 2 - 137 |
| Burns and Plastics | 460 | 4, 4-4 | 3 - 5 | 2 - 246 |
| Gynaecology | 294 | 3, 2-3 | 2 - 4 | 2 - 65 |
| Head and neck | 543 | 8, 8-9 | 4 - 15 | 2 - 84 |
| Orthopaedics | 1114 | 5, 5-5 | 3 - 9 | 2 - 140 |
| Spinal | 496 | 5, 4-5 | 3 - 8 | 2 - 67 |
| Thoracics | 1815 | 4, 4-4 | 3 - 6 | 2 - 181 |
| Urology | 2771 | 4, 4-5 | 3 - 7 | 2 - 151 |
| Vascular | 163 | 4, 4-5 | 3 - 7 | 2 - 43 |
| Total | 22218 | 6, 6-6 | 4 - 9 | 2 - 246 |

Table 6 Postoperative length of stay by DREAMing and component variables in the exploratory cohort

|  | N | Median, 95%CI  (days) | IQR (days) | Range (days) |
| --- | --- | --- | --- | --- |
| Drinking, eating, and mobilising | | | | |
| Yes | 13112 | 5, 5-5 | 3 - 7 | 2 - 246 |
| No | 9106 | 8, 8-8 | 6 -13 | 2 - 228 |
| Drinking, eating, mobilising, and IV fluids discontinued | | | | |
| Yes | 10348 | 4, 4-4 | 3 - 6 | 2 - 246 |
| No | 11870 | 7, 7-7 | 5 -12 | 2 - 228 |
| Drinking | | | | |
| Yes | 19487 | 5, 5-6 | 4 - 8 | 2 - 246 |
| No | 2731 | 10, 9-10 | 7 - 16 | 2 - 228 |
| Eating | | | | |
| Yes | 15302 | 5, 5-5 | 3 - 7 | 2 - 246 |
| No | 6916 | 8, 8-8 | 6 - 13 | 2 - 228 |
| Mobilising | | | | |
| Yes | 17207 | 5, 5-5 | 3 - 8 | 2 - 246 |
| No | 5011 | 8, 8-9 | 6 - 14 | 2 - 228 |
| IV fluids discontinued | | | | |
| Yes | 13411 | 5, 5-5 | 3 - 7 | 2 - 246 |
| No | 8807 | 8, 7-8 | 5 - 12 | 2 - 228 |

Table 7 Incidence of postoperative complications by DrEaMing status 24-hours after surgery. Composite pulmonary complications: requirement for respiratory support or treatment for suspected chest infection on day 7, or ≥ Grade II Clavien-Dindo moderate or severe respiratory complications at any time point. Composite cardiovascular complications: Hypotension requiring treatment, new acute coronary syndrome event, arrhythmia, or cardiogenic pulmonary oedema on day 7, or ≥ Grade II Clavien-Dindo cardiovascular complications at any time point. POMS gastrointestinal complications: Nausea, vomiting, abdominal distension, or inability to tolerate enteral diet.

|  | Exploratory cohort | |  | Colorectal subgroup | |
| --- | --- | --- | --- | --- | --- |
|  | DrEaMing | Not DrEaMing |  | DrEaMing | Not DrEaMing |
| Highest grade of postoperative complication (Clavien-Dindo) | | | | | |
| None | 8847 (67.6) | 4049 (44.9) |  | 2929 (67.2) | 1426 (49.1) |
| I | 2015 (15.4) | 1619 (17.8) |  | 684 (15.5) | 572 (19.4) |
| II | 1629 (12.4) | 2168 (23.8) |  | 541 (12.4) | 578 (19.5) |
| III-A | 284 (2.2) | 475 (5.2) |  | 93 (2.1) | 130 (4.4) |
| III-B | 223 (1.7) | 384 (4.2) |  | 91 (2.1) | 121 (4.1) |
| IV-A | 81 (0.6) | 282 (3.1) |  | 28 (0.7) | 72 (2.4) |
| IV-B | 20 (0.2) | 92 (1.0) |  | 8 (0.2) | 30 (1) |
| V | - | - |  | - | - |
| Composite pulmonary postoperative complication | | | | | |
|  | 244 (1.9) | 337 (3.7) |  | 67 (1.5) | 91 (3.1) |
| Composite cardiovascular postoperative complication | | | | | |
|  | 242 (1.9) | 437 (4.8) |  | 93 (2.1) | 130 (4.4) |
| Day 7 Postoperative morbidity survey (POMS) gastrointestinal complication | | | | | |
|  | 827 (6.3) | 1880 (20.7) |  | 422 (9.6) | 617 (21.0) |

Figure 2 Proportion of colorectal patients DrEaMing 24-hours after surgery by hospital. Hospitals submitting fewer than five cases were excluded


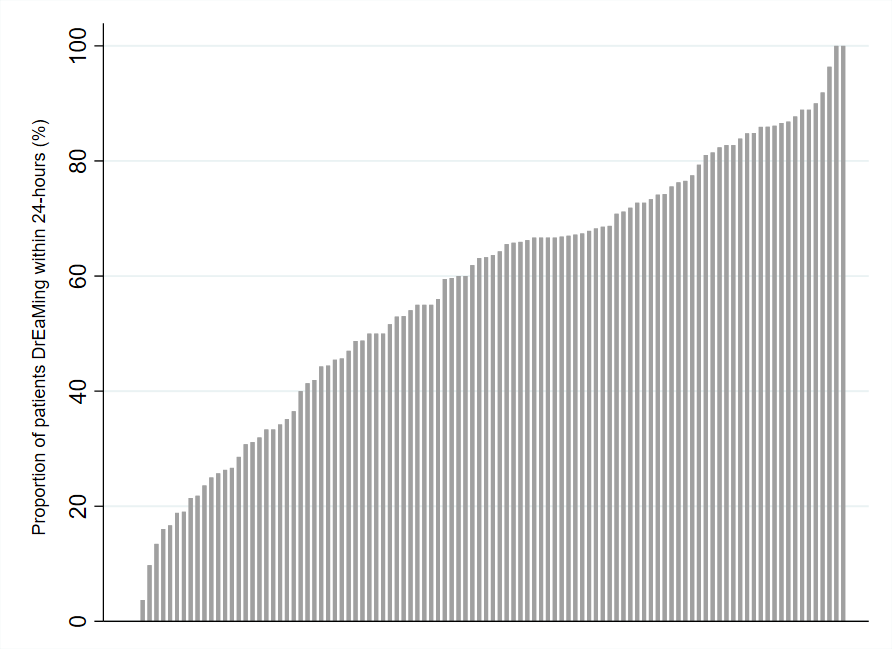


Table 8 postoperative length of stay by operative procedure in colorectal and multi-specialty cohorts

| Operative procedure | N= | Median, 95%CI  (days) | IQR (days) |
| --- | --- | --- | --- |
| Colorectal cohort | | |  |
| Anterior resection | 3200 | 6, 6-6 | 4 - 10 |
| Right hemicolectomy with anastomosis | 2955 | 6, 5-6 | 4 - 8 |
| Excision of sigmoid colon | 727 | 6, 6-6 | 4 - 10 |
| Left hemicolectomy with anastomosis | 348 | 6, 5-6 | 4 - 8 |
| Multi-specialty cohort | | |  |
| VATS lobectomy | 758 | 4, 4-4 | 3 - 6 |
| Total nephrectomy (non-transplant) | 741 | 4, 3-4 | 2 - 5 |
| Cystectomy | 673 | 8, 8-9 | 6 - 12 |
| Resection of lesion(s) of liver | 670 | 6, 6-6 | 4 - 8 |
| Pancreatoduodenectomy and excision of surrounding tissue (Whipple's procedure) | 594 | 10, 10-11 | 8 - 15 |
| Reversal of Hartmann's procedure | 523 | 6, 6-6.42 | 5 - 9 |
| Radical prostatectomy | 509 | 2, 2-3 | 2 - 4 |
| Revision of total replacement of knee joint | 427 | 5, 5-5 | 3 - 7 |
| Abdominoperineal (AP) resection with end colostomy | 417 | 8, 8-9 | 7 - 14 |
| Right hemicolectomy (with ileostomy) | 401 | 7, 6-7 | 5 - 11 |

Figure 3 Hospital-level variation in postoperative length of stay in the colorectal surgical cohort


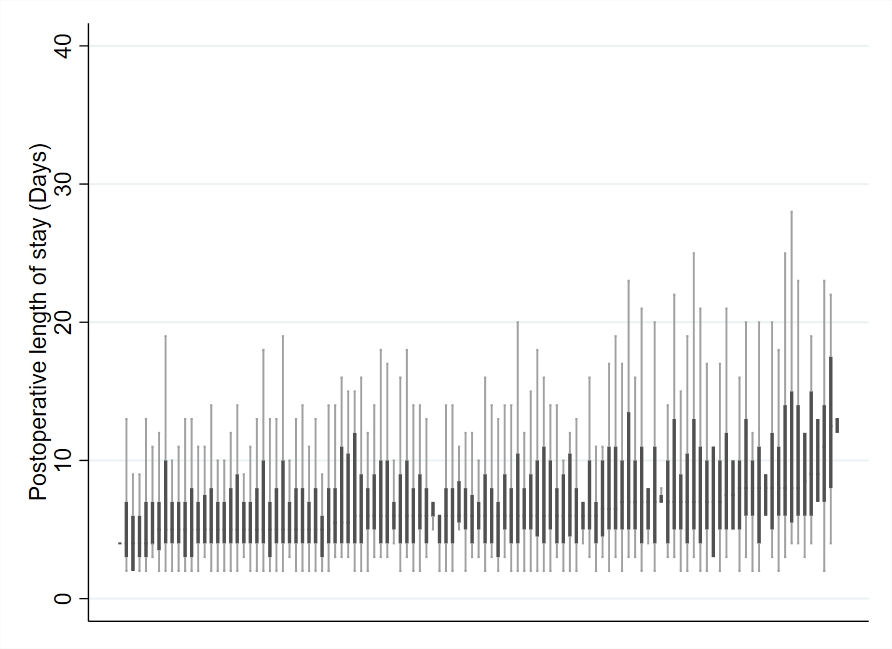


Table 9 postoperative length of stay by DrEaMing and component variables within 24-hours of surgery in colorectal cohort patients

|  | N | Median, 95%CI (days) | IQR (days) | Range (days) |
| --- | --- | --- | --- | --- |
| Drinking, eating, and mobilising | | | |  |
| Yes | 4341 | 5, 5-5 | 4-7 | 2 to 142 |
| No | 2889 | 7, 7-7 | 5-11 | 2 to 228 |
| Drinking, eating, mobilising, and IV fluids discontinued | | | |  |
| Yes | 3367 | 5, 5-5 | 4-7 | 2 to 61 |
| No | 3863 | 7, 7-7 | 5-11 | 2 to 228 |
| Drinking | | | |  |
| Yes | 6573 | 6, 6-6 | 4-8 | 2 to 142 |
| No | 657 | 8, 8-9 | 6-14 | 2 to 228 |
| Eating | | | |  |
| Yes | 4888 | 5, 5-5 | 4-8 | 2 to 142 |
| No | 2342 | 7, 7-7 | 5-12 | 2 to 228 |
| Mobilising | | | |  |
| Yes | 5925 | 5, 5-6 | 4-8 | 2 to 228 |
| No | 1305 | 8, 8-8 | 6-13 | 2 to 110 |
| IV fluids discontinued | | | |  |
| Yes | 4422 | 5, 5-5 | 4-7 | 2 to 72 |
| No | 2808 | 7, 7-7 | 5-11 | 2 to 228 |

Table 10 Predictors of prolonged postoperative LOS after multispecialty surgery: multilevel analysis testing DrEaMing as the variable of interest (Median odds ratio 0.43 (0.32-0.58) p<0.001). *: POSSUM or combined POSSUM categories, ECG: electrocardiograph, AF: atrial fibrillation

| Variable | | Odds Ratio | 95% CI | p |  | | | | | | | | | | Variable | | | | Odds Ratio | 95% CI | | p | |  |
| --- | --- | --- | --- | --- | --- | --- | --- | --- | --- | --- | --- | --- | --- | --- | --- | --- | --- | --- | --- | --- | --- | --- | --- | --- |
| Variable of interest | | | | |  | | | | | | | | | | Perioperative processes and metrics | | | | | | |  | |  |
| DrEaMing status at 24-hours | | | | |  | | | | | | | | | | Surgical specialty | | | | | | |  | |  |
|  | Delivered | 0.53 | 0.45 - 0.63 | <0.001 |  | | | | | | | | | |  | | Abdominal - other | | 0.28 | 0.05 - 1.52 | | 0.14 | |  |
|  | Not delivered | Ref |  |  |  | | | | | | | | | |  | | Hepatobiliary | | 0.38 | 0.27 - 0.53 | | <0.001 | |  |
| Casemix variables | | | | |  | | | | | | | | | |  | | Lower gastrointestinal | | Ref |  |  |  | |  |
| Preoperative physiological and biochemical variables | | | |  |  | | | | | | | | | |  | | Orthopaedic | | 1.25 | 0.80 - 1.95 | | 0.33 | |  |
|  | Age (Years) | 1.03 | 1.02- 1.03 | <0.001 |  | | | | | | | | | |  | | Thoracic | | 1.87 | 1.29 - 2.70 | | <0.001 | |  |
|  | Body mass index | 1.00 | 0.99- 1.02 | 0.70 |  | | | | | | | | | |  | | Upper gastrointestinal | | 0.10 | 0.02 - 0.47 | | <0.001 | |  |
|  | Serum [sodium] | 0.98 | 0.96- 1.01 | 0.13 |  | | | | | | | | | |  | | Urology | | 1.28 | 1.01 - 1.64 | | 0.05 | |  |
|  | Serum [potassium] | 1.24 | 1.03- 1.48 | 0.02 |  | | | | | | | | | | Number of preceding operations during this admission | | | | | | |  | |  |
|  | Serum [creatinine] | 1.00 | 1.00- 1.01 | 0.24 |  | | | | | | | | | |  | | None | | Ref |  | |  | |  |
|  | Serum white cell count | 1.01 | 0.98- 1.04 | 0.37 |  | | | | | | | | | |  | | ≥1 | | 1.34 | 0.97 - 1.85 | | 0.08 | |  |
|  | Serum [Haemoglobin] | 0.96 | 0.91- 1.00 | 0.06 |  | | | | | | | | | | Mode of surgery | | | |  |  | |  | |  |
|  | Heart rate | 1.00 | 0.99- 1.01 | 1.00 |  | | | | | | | | | |  | | Open | | Ref |  | |  | |  |
|  | Systolic blood pressure | 1.00 | 0.99- 1.00 | 0.47 |  | | | | | | | | | |  | | Laparoscopic/ robotic | | 0.54 | 0.45 - 0.65 | | <0.001 | |  |
|  | Oxygen saturations | 0.97 | 0.92- 1.02 | 0.23 |  | | | | | | | | | | Enhanced recovery protocol | | | | |  | |  | |  |
| Sex | |  |  |  |  | | | | | | | | | |  | | Used | | 0.79 | 0.65 - 0.95 | | 0.01 | |  |
|  | Male | 1.07 | 0.90- 1.27 | 0.47 |  | | | | | | | | | |  | | Not used | | Ref |  | |  | |  |
|  | Female | Ref |  |  |  | | | | | | | | | | Preoperative bowel preparation administered | | | | |  | |  | |  |
| ASA-PS | |  |  |  |  | | | | | | | | | |  | | Yes | | 0.77 | 0.61 - 0.97 | | 0.03 | |  |
|  | 1 | Ref |  |  |  | | | | | | | | | |  | | None | | Ref |  |  |  | |  |
|  | 2 | 1.29 | 0.93- 1.79 | 0.13 |  | | | | | | | | | | Preoperative carbohydrate administered | | | | |  | |  | |  |
|  | 3 | 1.51 | 1.06- 2.15 | 0.02 |  | | | | | | | | | |  | | Yes | | 0.94 | 0.77 - 1.15 | | 0.54 | |  |
|  | 4 or 5 | 2.02 | 0.92- 4.41 | 0.08 |  | | | | | | | | | |  | | Unknown | | 0.97 | 0.77 - 1.21 | | 0.77 | |  |
| Malignancy* | | |  |  |  | | | | | | | | | |  | | None | | Ref |  | |  | |  |
|  | No solid tumour | Ref |  | |  | | | | | | | | | | Perioperative epidural analgesia | | | | |  |  |  | |  |
|  | Local disease | 0.85 | 0.68- 1.05 | 0.14 |  | | | | | | | | | |  | | Yes | | 1.46 | 1.17 - 1.82 | | <0.001 | |  |
| Nodal or metastatic spread | | 1.07 | 0.82- 1.40 | 0.60 |  | | | | | | | | | |  | | None | | Ref |  |  |  | |  |
|  |  |  |  | |  | | | | | | | | | | Intrathecal analgesia | | | | 1.15 | 0.94 - 1.40 | | 0.16 | |  |
| Preoperative ECG* | |  |  | |  | | | | | | | | | |  | | Yes | |  |  | |  | |  |
|  | No abnormality | Ref |  | |  | | | | | | | | | |  | | None | | Ref |  | |  | |  |
|  | AF: rate 60-90 bpm | 0.79 | 0.53- 1.17 | 0.24 |  | | | | | | | | | | Regional anaesthesia/ analgesia | | | | |  |  |  | |  |
|  | AF >90 / other abnormality | 0.91 | 0.73- 1.14 | 0.41 |  | | | | | | | | | |  | | Yes | | 0.90 | 0.71 - 1.13 | | 0.37 | |  |
|  | ECG was not performed | 1.43 | 1.04- 1.95 | 0.03 |  | | | | | | | | | |  | | None | | Ref |  |  |  | |  |
| Cardiac findings* | |  |  | |  | | | | | |  | | | | Intraoperative depth of anaesthesia monitoring | | | | | | |  | |  |
|  | No failure | Ref |  | |  | | | | | |  | | | |  | | Used | | 0.87 | 0.71 - 1.05 | | 0.15 | |  |
|  | Diuretic/ antihypertensive | 1.05 | 0.88- 1.25 | 0.61 |  | | | | | | | | | |  | | Not used | | Ref |  |  |  | |  |
|  | Peripheral oedema/ cardiomegaly | 1.42 | 0.86- 2.33 | 0.17 |  | | | | | | | | | | Intraoperative neuromuscular monitoring | | | | |  | |  | |  |
| Dyspnoea* | |  |  | |  | | | | | |  | | | |  | | Used | | 1.12 | 0.94 - 1.33 | | 0.22 | |  |
|  | None | Ref |  | |  | | | | | |  | | | |  | | Not used | | Ref |  |  |  | |  |
|  | On exertion | 1.19 | 0.95- 1.49 | 0.14 |  | | | | | | | | | | Intraoperative temperature probe use | | | | |  | |  | |  |
|  | Limiting exertion/ at rest | 1.34 | 0.90- 2.02 | 0.15 |  | | | | | | | | | |  | | Used | | 1.03 | 0.84 - 1.26 | | 0.78 | |  |
| NYHA class | |  |  | |  | | | | | |  | | | |  | | Not used | | Ref |  |  |  | |  |
|  | I | Ref |  | |  | | | | | |  | | | | Duration of surgery | | | |  |  |  |  | |  |
|  | II | 0.94 | 0.75- 1.17 | 0.57 |  | | | | | | | | | |  | | <2 hours | | Ref |  |  |  | |  |
|  | III or IV | 1.10 | 0.69- 1.76 | 0.69 |  | | | | | | | | | |  | | 2-3 hours | | 1.33 | 0.87 - 2.02 | | 0.18 | |  |
| Cerebrovascular disease | |  |  | |  |  | |  | | | |  | | | |  | | >3 hours | 1.72 | 1.14 - 2.59 | | 0.01 | |  |
|  | History | 1.14 | 0.82- 1.59 | 0.45 |  | | | | | | | | | | Postoperative level of care | | | | |  |  |  | |  |
|  | No history | Ref |  | |  |  | |  | | | |  | | | |  | | 0 | Ref |  |  |  | |  |
| Dementia | |  |  | |  | |  | |  | | | |  | |  | | 1 | | 1.49 | 1.13 - 1.96 | | | 0.01 | |
|  | History | 1.46 | 0.62- 3.45 | 0.39 |  | | | | | | | | | |  | | 1.5 or 2 | | 1.56 | 1.26 - 1.94 | | <0.001 | |  |
|  | No history | Ref |  | |  | |  | | |  | | | |  |  | | 3 | | 1.89 | 1.30 - 2.74 | | <0.001 | |  |
| Diabetes mellitus | |  |  | |  | |  | | |  | | | |  | Severity of postoperative pain in recovery | | | | |  | |  | |  |
|  | None | Ref |  | |  | |  | | |  | | | |  |  | | None | | Ref |  |  |  | |  |
|  | Non-insulin dependent | 1.15 | 0.92- 1.45 | 0.22 |  | | | | | | | | | |  | | Mild | | 1.19 | 0.98 - 1.45 | | 0.08 | |  |
|  | Insulin dependent | 1.06 | 0.74- 1.51 | 0.76 |  | | | | | | | | | |  | | Moderate | | 1.11 | 0.90 - 1.36 | | 0.34 | |  |
| Liver disease | |  |  | |  | |  | | |  | | | |  |  | | Severe | | 1.17 | 0.90 - 1.52 | | 0.23 | |  |
|  | History | 1.81 | 1.03- 3.18 | 0.04 |  | | | | | | | | | |  | | Unable to assess | | 1.28 | 0.94 - 1.75 | | 0.12 | |  |
|  | No history | Ref |  | |  | |  | | |  | | | |  | Core temperature >36°C in recovery room | | | | |  | |  | |  |
| Smoking history | |  |  | |  | |  | | |  | | | |  |  | | Yes | | 0.96 | 0.76 - 1.21 | | 0.71 | |  |
|  | Never smoked | Ref |  | |  | |  | | |  | | | |  |  | | No | | Ref |  |  |  | |  |
|  | Unknown | 0.89 | 0.62- 1.29 | 0.55 |  | | | | | | | | | | Intra-abdominal drain in place postoperatively | | | | |  | |  | |  |
|  | Quit >6 months ago | 0.99 | 0.83- 1.17 | 0.88 |  | | | | | | | | | |  | | Yes | | 0.97 | 0.80 - 1.18 | | 0.78 | |  |
|  | Quit <6 months ago | 0.93 | 0.65- 1.33 | 0.69 |  | | | | | | | | | |  | | None | | Ref |  |  |  | |  |
|  | Current smoker | 1.14 | 0.88- 1.46 | 0.32 |  | | | | | | | | | | Nasogastric tube in place postoperatively | | | | |  | |  | |  |
| Operative urgency | | |  | |  | |  | | |  | | | |  |  | | Yes | | 1.10 | 0.86 - 1.40 | | 0.47 | |  |
|  | Elective | Ref |  | |  | |  | | |  | | | |  |  | | None | | Ref |  |  |  | |  |
|  | Expedited | 0.94 | 0.71- 1.23 | 0.63 |  | | | | | | | | | | Day of the week surgery was performed | | | | |  | |  | |  |
| Major postoperative complication(s) | | |  | |  | |  | | |  | | | |  |  | | Monday | | 1.11 | 0.89 - 1.40 | | 0.36 | |  |
|  | ≥1 | 6.72 | 5.71- 7.90 | <0.001 |  | | | | | | | | | |  | | Tuesday | | Ref |  |  |  | |  |
|  | None | Ref |  | |  | |  | | |  | | | |  |  | | Wednesday | | 1.36 | 1.09 - 1.70 | | 0.01 | |  |
|  |  |  |  | |  | |  | | |  | | | |  |  | | Thursday | | 1.07 | 0.85 - 1.35 | | 0.55 | |  |
|  |  |  |  | |  | |  | | |  | | | |  |  | | Friday | | 1.08 | 0.84 - 1.40 | | 0.53 | |  |
|  |  |  |  | |  | |  | | |  | | | |  |  | | Saturday | | 0.79 | 0.27 - 2.32 | | 0.67 | |  |
|  |  |  |  | |  | |  | | |  | | | |  |  | | Sunday | | 1.72 | 0.64 - 4.66 | | 0.28 | |  |
