# Supplementary material for: Delivery of drinking, eating and mobilising (DrEaMing) and its association with length of hospital stay after major noncardiac surgery: observational cohort study
Source: Br J Anaesth. 2022 May 12;129(1):114–26. doi: 10.1016/j.bja.2022.03.021 (PMC9284668; doi:10.1016/j.bja.2022.03.021)
Supplement: Multimedia component 2 [file mmc2.pdf]

PQIP COLLABORATIVE

Mayavan Abayalingam
Sue Abdy
Andrea Ackerman
Ian Adams
David Adamson
Anjum Ahmed-Nusrath
Doug Aitken
Louise Akeroyd
Nick Aldridge
Tamara Alexander
Hind Al-Husain
Syed Ali
Jason Ali
Charlotte Allan
Laurin Allen
Wei-Lin Allen
Denise Almond
Ziad Alrifai
Edward Andrade
Gillian Ansell
Gill Arbane
Giuseppe Aresu
Susanne Armitage
Glenn Arnold
Jon Arnot-Smith
Linda Ashley–Edden
Anam Asif
Sheila Avery
Jennifer Awolisi
Ewa Babisz
Morgan Back
Madhu Balasubramaniam
Samantha Banks
Rebecca Barker
Veronica Barnes
Gillian Barnett
Nina Barratt
Andy Bates
Rachel Baumber
Rose Beesley
Martha Belete
Jane Bellamy
Melissa Benavente
Julia Benham-Hermetz

Eva Benke
Jenny Bennett-Britton
Anna Bewlay
Chetan Bhan
Ravi Bhatia
Arnab Bhowmick
Sian Birch
Brian Bisase
Kevin Blizzard
Aneta Bociek
Clare Bolger
Pieter Bothma
Anne-Marie Bougeard
Rebecca Boulton
Andy Bracewell
Joanne Bradley-Potts
Elizabeth Bradshaw
Debbie Branney
Elizabeth Brannigan
Philip Braude
David Brealey
Andrew Brennan
Anne-Jayne Brien
Kathryn Brodbelt
Helen Bromhead
Rachel Burnish
Wendy Byrne
Debbie Campbell
Debra Campion
Sara Campos
Joel Cardoso-Pereira
Sue Carnell
Ben Carrick
Clarissa Carvalho
Ben Chandler
Mogera Chandra
Asif Chaudry
Irfan Chaudry
Belinda Chieng
Mahindra Chincholkar
Sadasivan Chinniah
Maria Ciaponi
Emma Clarey
Richard Clark
Tom Clark
Edwin Clark

Sarah Clarke
Hannah Clarke
Tristan Clarke
Peter Coe
Helen Cole
Libby Cole
Julie Colley
Nikki Collings
Clare Conlon
Karen Connolly
Daniel Conway
Sarah Cooper
Karen Copeland
Zoe Copp
James Corcoran
Victoria Cordrey
Henry Corner
Darryl Cornish
James Coulston
Nicholas Courtenay-Evans
James Craig
Jayne Craig
Jason Cross
Janette Curtis
Claire Dalton
Diane Daniels
Roger Davies
Jenny Davis
Khaled Dawas
Peter Delve
Mohammed Deputy
Amit Deshmukh
Somi Desikan
Jugdeep Dhesi
Matt Dickinson
Toby Dixson
Emma Dougherty
Claire Dowse
Steve Drage
Dragos Dragnea
Kate Driver
Stephen Duberley
Lucy Dudgeon
Sarah Duff
Stephanie Dukes
Lauren Duraman

Rebecca Dyar
Simon Dyer
Jonathan Easterbrook
Christine Eastgate
Mark Edwards
Ramesh Ekambaram
Kerry Elliott
Serena Elliott
Ahmed El-Sharkawy
Mark Eltringham
Jonathan Epstein
Matt Everson
Mark Fairbrass
Joanne Falconer
Sarah Farnell-Ward
Naila Farooq-Iqbal
Helen Farrah
Catherine Farrow
Imogen Fecher
Teresa Ferreira
Helder Filipe
Stuart Finn
Gracie Fisk
Jill Fitchett
Katie Flower
Jessica Forrester
Damon Foster
Ming Fung
Jackie Furlong
Rosie Furness
Claire Gallagher
Fang Gao Smith
James Garrod
Emma Gent
Norma Gibbons
Kayleigh Gilbert
Julian Giles
Marc Gimenez
Yvonne Gleeson
Kelly Goffin
Dhanny Gomez
Gayna Grantham
Juan Graterol
Irina Grecu
Louise Greig
Lisa Grimmer

Chris Grocock
Mike Grocott
Esra Gumus Bayazit
Paul Gunning
Denise Hadfield
Chandima Halahakoon
George Hallward
Sarah Hammons
Vivienne Hannon
Clair Harris
Colin Harris
Robert Hartley
David Harvie
Jess Heaps
Sarah Herbert
Emily Hetherington
Matt Hill
Ned Hobbs
Tim Hodgkins
Sheila Hodgkinson
Tracy Hodgkiss
James Holding
Liz Hood
Kathleen Horan
Deborah Horner
Tanvir Hossain
Tim Hughes
Jenny Hughes
Alistair Hughes
Rob Hull
Spencer Humphrys
Louise Hunt
Lisa Hurley
Melanie Hutchings
Mark Ibrahim
Ugo Ihedioha
Jennifer Illingworth
Matthew Jackson
Reni Jacob
Vipul Jain
Kate James
Nalayini Jeyavel
Shaman Jhanji
Helgi Johannsson
Joanne Johnson
Carolyn Johnston

Dawn Johnstone
Cathy Jones
Laura Abernathy
Nkemjika Abiakam
Oluronke Adanini
Maame Aduse-Poku
Neil Agnew
Michael Agyemang
Salman Ahmad
Sarfraz Ahmad
Lindianne Aitken
Mansoor Akhtar
Timothy Alce
Rod Alexander
Bilal Alkhaffaf
Clare Allcock
Jo Allison
Elana Anastasescu
Prematie Andreou
Eleanor Andrews
George Antoniou
Michael Argent
Nadeem Ashraf
Omer Aziz
Sheeba Badu
Craig Bailey
Tessa Bailey
Pearl Baker
Mansoor Bangash
Smita Bapat
Chris Barben
Musa Barkeji
Debi Barnes
Lisa Barrell
James Barrowman
Katherine Batte
Hannah Beadle
Chloe Beard
Paul Bedford
Kate Beesley
Xiao Bei Zhao
Jan Belcher
Sarah Bell
Jocelyn Bell
Gillian Bell
Zoe Bennettan

Mariana Bernardo
Stephanie Berry
Emily Bevan
Kailash Bhatia
Edward Bick
Janine Birch
Janine Bird
Ethel Black
Lauren Blackburn
Verity Blackburn
Doug Blackwood
Jackie Bladon
Melanie Bloor
Andrea Boedo
Jessica Bowen
Sophie Boyd
Sharon Boyne
Andrew Bracewell
Julie Bracken
John Bramall
Barbara Bray
Matthew Bridge
Elaine Brinkworth
Catherine Britton Jones
Fiona Brogan
Adam Brooks
Johanna Brown
Michelle Bruce
Zoe Brummell
Sarah Buckley
Rhian Bull
Karen Burns
Hazel Cahill
David Cain
Bridget Campbell
Marta Campbell
Suzanne Carey Jones
Millie Carnachan
David Carter
Jaime Carungcong
Paula Carvelli
Kim Chan
Tristan Chapman
Katy-Jane Chick
Anna Chillingworth
Gayathri Chinnappa Srinivas

Sara Churchill
Amy Clark
Thomas Clark
Sheron Clarke
Leon Cohen
Martin Cole
Andrea Cole
Alison Colhoun
Peter Collett
Dawn Collier
Rachel Collis
Thomas Collyer
Edward Combes
Louise Conner
Tim Cook
Nadine Cooper
Lisa Cooper
Jim Corcoran
Patricia Correia da Costa
Edward Costar
Edward Courtney
Amanda Cowton
Nicholas Crabtree
Andrew Cresswell
Nikki Crisp
Jennifer Crooks
Carina Cruz
Peter Csabi
Fiona Culley
Zoe Daly
Sarah Daniels
Thelma Darian
Bobby Dasari
Dave Smith
Beena David
Louisa Davies
Amanda Davies
Charlie Davies
Richard Davies
Samantha Davies
Andrew Day
Toni de Freitas
Joanne Deery
Anne Devine
Priya Dias
Chimverly Diaz

Lisa Ditchfield
Hiren Divecha
Annette Dowdell
Ronald Druyeh
Leon Dryden
Steven Dunkley
Almu Duran-Rosa
Rebecca Dyer
James Eales
Megan Eardly
Tracy Edmunds
Emma Edmunds
Mandy Edwards
Ashley Elden
Kaytie Ellis
Iony Evans
Paul Evans
Fiona Fitzgerald
Jayne Foot
Selina Ford
Greg Forshaw
Kito Fusai
Claire Gabriel
Joanne Galliford
Carina Galpin
Amina Garcia
Mark Garfield
Zoe Garland
Theresa Garratt
Philip Gedling
Emma Gendall
Roman Genetu
Simon George
Ranjit Gidda
Kim Giraud
Jon Glass
Prisca Gondo
Romana Govindaraju
Lia Grainger
Neus Grau Novellas
Caris Grimes
Jessica Gunn
Zoe Guy
Laura Gwathil
John Gwatkin
Brigid Hairsine

Bence Hajdu
Dawn Hales
Angela Hall
Geraldine Hambrook
Vincent Hamlyn
Kate Hammerton
Sarah Hammond
Fiona Hammonds
Aimee Hampton
Janice Hanley
Daniel Hanratty
Will Hare
Daniel Harper
Andrea Harren
Yasmin HarringtonDavies
Stephen Harris
John Harris
Sanjay Harrison
Alister Hart
Emma Hartland
Liz Hawes
Nancy Hawkins
Christine Hawkins
Kat Haynes
Olivia Hayward
Chantelle Heppolette
Sarah Hierons
Susan Hill
Simon Hindley
Elizabeth Hodge
Tracey Hodgkiss
Gemma Hodgson
Maureen Holland
Ann Holmes
Cassandra Honeywell
Anil Hormis
Kate Howard
Linda Howard
Georgina Howell
Lucie Howie
Jo Hubert
Danielle Huckle
Karen Hudson
Rachel Hughes
Adam Hunt
Nik Husain

Arjuna Imbuldeniya
Fiona Ingoldby
Francious Ioffidis
Jacqueline Routledge
Zara Jalal
Rose Jama
Sunil Jamadarkhana
Katherine James
Parandeep Jandu
Anna Janowicz
Anne Jayne Brien
Richard Jennings
Emily Jeynes
Mhairi Jhugursing
Jo Bennett
Teishel Joefield
Summayyah Jogi
Rebekah Johnson
Linda Johnson
Rob Johnston
Rhidian Jones
Claudette Jones
Lyndon Jones
Colette Jones-Criddle
Lesley Jordan
Matt Julian
Miriam Kadry
Prashant Kakodkar
Vidya Kasipandian
Ramanathan
Kasivisvanathan
Tasneem Katawala
Jasmin Kaur
Sharon Kaur
Ambreen Kauser
Anne Keen
Natalie Keenan
Jessica Kelly
Andrea Kelly
Peter Keltie
Clive Kelty
Christopher Kennedy
Jo Keogh
Amy Kerr
Joyce Kibaru
Katie King

Maureen King
Jane Kingham
Harry Knight
John Knight
Peter Knowlden
Shum Koin Lon
Dimitrios
Konstantopoulos
Christos Kontovonisious
Damir Kosutic
Kartik Kota
Bobby Krishnachetty
Priya Krishnan
Monika Krupa
Agniezsa Kubisz-Pudelko
Mohan Kumar
Vikas Kumar
Snehal Kumbhare
Rajeev Kushwaha
Inese Kutovaja
Thyra Kyere-Diabour
James Lai
Sandeep Lakhani
Aroon Lal
Jo Lambert
Ben Lane
Tamsin Lane
Katy Later
Maurice Lau
Helen Lawrence
Tessa Lawrence
Heidi Lawson
William Lawson-Brown
Tom Lawton
Susannah Leaver
Julie Lebas
Gemma Lee
Michelle Leemans
Mairi Leggatt
Cynthia Leigh
Dee Leonard
David Leslie
Denny Levett
Christopher Levett
David Levy
Juliette Li Wan Po

Rosario Lim
Woei Lin Yap
Jonathan Lloyd Evans
Alison Loftus
Ruth Longfellow
Rita Lopes
Rui Lopes
Bruno Lorenzi
Gretel Loten
Robert Loveridge
Justine Lowe
Clare Lummis
Val Luoma
Craig Lyness
Marc Lyons
Sara Ma
Neil MacDonald
Mark MacGregor
Ami Mackay
Ailie Mackenzie
Jennifer MacLellan
Joe Macmillan
George Madden
Karen Maher
Parisa Mahjoob-Afag
Christopher Mahon
Cathryn Mainwaring
Sola Makinde
Jasmina Mandair
Mira Manoharan
Hosnieh Marbini
Kalimuthu Marimuthu
Nicky Marks
Suzie Marriott
Emma Marshall
Jane Martin
Tim Martin
Sarah Martindale
Guillermo Martinez
Laura Martins
Gladys Martir
Sushil Maslekar
Clare Mason
Alexandra Matson
Rosanna Maurin
Debbie Mawson

John May
Denzil May
Frank McAuley
Laura McCafferty
Ananya McCarthy
Jacqueline Mccormick
Bruce McCormick
Richard McCormick
David McCrettton
Evanna McEvoy
John McGrath
Sophie McGrath
India McKenley
Emma Mckenna
Elizabeth McKerrow
Euan McLaughlin
Laura Mcleavy
Fiona McNeela
Margaret McNeil
Denise McSorland
Johannes Mellinghoff
Teresa Melody
Julie Melville
Arun Menon
Stuart Mercer
Pauline Mercer
Megan Meredith
Susan Merotra
Rachel Meskell
Clare Mewies
Maria Milburn
Martin Millar
Jessica Miller
Don Milliken
Simran Minhas
Gary Minto
Sarah Mitchard
George Mochloulis
Jignasa Modha
Faizal Mohomed-Hossen
Helen Molloy
Georgia Monantera
Rugaia Montaser
Jane Montgomery
Maria Moon
Claire Moore

Tim Moore
Alison Moore
Iain Moppett
Lucy Moran
Tom Morgan-Jones
Catherine Moriarty
Clare Morkane
Rebecca Morris
Lucy Morris
Sophie Morris
Andy Morrison
Alison Moss
Susan Moss
Bhvani Mothe
Lorraine Motuel
Jessica Muchmore
Karim Muhammad
Hassan Mukhtar
Amanda Mulholland
Joe Mullender
Amanda Mullholland
Maxene Murdoch
Henry Murdoch
Rosie Murdoch
Jo Murfin
Anthony Murphy
Zoe Murphy
Katherine Murray
Dave Murray
Rosemary Musanhu
Esther Mwaura
Priya Nadarajah
Seema Nadkarni
Zin Naing
Rajesh Nair
Ashok Nair
Priya Nair
Shireen Naqui
Andres Naranjo
Nagendra Natarajan
Noel Nathaniel
Shakira Nathoo
Deanna Naylor
Aaron Ng
Yang Ng
Onie Ngwenya

Sotiris Nicholas
Claire Nicholas
Tom Nightingale
Louise Nimako
Marie Nixon
Hannah Noble
Harriet Noble
Aidan Noon
Julie North
Kribashnie Nundlall
Ruth O'Dowd
Paul O'Loughlin
Vikki O'Loughlin
Jonathan Ockrim
Tessa O'Halloran
Maxine Okello
Alison O'Kelly
Padraig O'Scannill
Ayo Oshowo
Harriet Owen
Sara Owen
Jamie Pack
Andrew Padwick
Valerie Page
Katherine Pagett
Anirudda Pai
Glykeria Pakou
Carole Paley
Claire Palmer
Janet Palmer
Shivani Pandya
Kerry Paradowski
Dhruv Parekh
Zarah Paris
Linda Park
Jane Parker
David Parkinson
Valerie Parkinson
Sanjay Parmar
Julia Parnell
James Parry
Penny Parson
Georgie Parsons
Judith Partridge
Jonathan Pass
Jaimin Patel

Rajan Patel
Johanna Paterson
Kate Paterson
Abigail Patrick
Mathew Patteril
Dan Paul
Mark Paul
Varghese Paul
Mark Pauling
Stephanie Pauling
Nikhil Pawa
Corinne Pawley
Louise Pearson
Andrew Peethamsingh
Suzannah Pegler
Melchizedek
Penacerrada
Lisa Penny
Stacey Pepper
Lauren Perkins
Raj Pervalli
Cecilia Peters
Chris Peters
Carroll Petty
Alexander Philips
Andrew Pick
Mikolaj Pielas
Manuel Pinto
Lucy Pippard
Bala Piramanayagam
James Plumb
Kathryn Pointon
Sara Polhill
Michael Pollard
Fiona Pomeroy
Jim Poncia
Mel Poole
Claire Potter
Alison Potter
Gail Pottinger
Sarah Powell
Chris Powell-Wiffen
Oliver Pratt
Joel Prescott
Karen Prevc
Anna Price

Carly Price
Stephanie Prince
Florence Prior
Ffion Pritchard
Mark Pulletz
Anne Pullyblank
Bally Purewal
Charlotte Quamina
Ramasamy Radhika
Govindaraju Ramana
Sean Ramcharan
Lidia Ramos
Nirmalabaye Ramsamy
Fiona Ramsden
Simon Rang
Mohan Ranganathan
Valluvan Rangasamy
Sameer Ranjan
Rajashankar Rao
Steph Ratcliffe
Dave Raw
Shilpa Rawat
Caroline Reavley
Jon Redman
Ellie Reeves
Hafiz Rehman
Karen Reid
Simon Reid
Andrew Renehan
Johannes Retief
Adam Revill
Nicolas Rey de Castro
Anna Reyes
Ramasamy Rhadika
Matthew Rhodes
Karen Rhodes
Anna Riccoboni
Zoe Ridgway
Stephanie Ridgway
Corinne Rimmer
Jenny Ritzema
Vanessa Rivers
Stephen Roberts
Martyn Robertson
Nikola Robinson
Lisa Roche

Kirsty Rogers
Melissa Rosbergen
Alastair Rose
Joanne Rothwell
Geena Roy
James Royal
Anna Roynon Reed
Webster Rushesha
Lucy Ryan
Christine Ryan
Parv Sains
Amina Sajid
Mark Salmon
Collette Samuels
Amanda Sanderson
Siva Sangaralingham
Sumayer Sanghera
Seliat Sanusi
Laura Sarmiento Valero
Christine Sathananthan
Nicholas Savage
Heather Savill
Amrinder Sayan
Magda Sbai
Andrea Scala
Mark Scarfe
Rosaria Scarpinata
Lyndsay Scarratt
Anne Scase
Louise Schonborn
Simon Scott
Michaela Scott
Chloe Searles
Karthikeyan Selvaraju
Neel Sengupta
Victoria Senior
Darreul Sewell
Helen Seymour
Nirav Shah
Samir Shah
Deep Shah
Andy Shannon
Sophie Shapter
Emma Sharkey
Helen Sharples
Adnan Sheikh

Tom Sheppard
Julie Sheriff
Paula Shirley
Anthony Short
Charmaine Shovelton
Pauline Sibley
Constantinos Simillis
Helen Simmons
Joanna Simpson
Janet Sinclair
Jambulingam Sivasamy
Subash Sivasubramaniam
John Skinner
Amy Slack
Kirstie Smith
Tim Smith
Chris Smith
Darren Smith
Austen Smith
Julian Smith
Jason Smith
Rachel Smith
Neil Smith
Theresa Smith
Jennifer Smith
Fran Smith
Debbie Smyth
Rebecca Snell
Manisha Sodhi
Kathryn Sollesta
Julian Sonksen
Simon Sparkes
William Speake
Will Spencer
Yolande Squire
Gemma Squires
Philippa Squires
Seema Srivastava
Frank Stafford
Claire Stapleton
Lorraine Stephenson
Joseph Stevens
Nathalie Stevenson
Richard Stewart
Duncan Stewart
Adrienne Stewart

Julian Stone
Mark Stoneham
Sharon Storton
Alexa Strachan
Richard Struthers
Charlotte Strzelecki
Daren Subar
Akshay Sule
Mark Sullivan
Jaysimha Susarla
Paul Sutton
Asheesh Suxena
Andrew Swain
Catherine Swann
Kathy Swanson
Mike Swart
Katie Sweet
Yadullah Syed
Abdul Syed
Rebecca Symes
Gemma Szabo
Kata Szabo
Melanie Tan
James Taylor
Frances Taylor
Michelle Taylor
Natalie Taylor
Emma Temlett
Nila Tewari
Azeem Thahir
SriKandan Thangavel
Mini Thankachen
Bubby Thava
Abrie Theron
Kumaran Thiruppathy
Kannan Thogulava
Caroline Thomas
Sue Thomas
Vicky Thomas
Rebecca Thompson
Leah Thompson
Chris Thorn
Madeleine Thyssen
Helen T-Michael
Gabriella Tomkova
Zara Townley

Dawn Trodd
Maria Troy
Julia Tubbs
Olga Tucker
James Tulloch
Victoria Turner
Ian Turner-Bone
Cadice Tyers
David Tyl
Stephen Usher
Chandrashekhar
Vaidyanath
Chandra Vaidyanath
Luke Vamplew
Emma Varley
Nikhil Vasdev
Rajiv Vashisht
Frances Venn
Joanne Vere
Mark Vertue
Julie Vickery
Dale Vimalachandran
Marcela Vizcaychipi
Ravinder Vohra
Lewis Waggett
Mai Wakatsuki
Jessica Walding
Susanna Walker
Elaine Walker
James Walkington
Deirdre Wallace
Jo Waller
Lucy Walsh
Michelle Walter
Daniel Wang
Sinead Ward
John Ward
Sally Ward-Booth
Gregory Warren
Tim Warrener
Richard Wassall
Lucy Waterfield
Grant Watling
Jane Watson
Nicholas Watson
Dave Watson

Philip Waugh
Emily Weaver
Jonathan Williamson
Matthew Whitehead
Danielle Wilcock
Laura Wilding
Emma Wiliams
Gail Williams
Hannah Wilson
Jonathan Wilson
Danny Wong
Andrew Woodgate
Jade Woolley
Caroline Wrey Brown
Retno Wulandari
Babel Zaheer
Ahmad Ziyad
Barbara Bray
David Carter
Gayathri Chinnappa
Srinivas
Nicholas CourtenayEvans
Yasmin Harrington-Davies
John Harris
Melchizedek Penacerrada
Pippa Squires
Seema Srivastava
Miriam Davey
Beth Jones
Jamie Goodman
Helen Sankey
Michelle Page
Alex Matson
Jamie Irisari
Mark Priestly
Miranda Baum
Susan Kelly
Rebecca Kanu
Roxana Juncu
Darylile Guledew
Katherine Mackintosh
Stephen Webb
Poonam Bopanna-Muckatira
Owen Lewis
Sean Cutler
Maxine Nash

Lynn Wren
Sara-Beth Sutherland
Sonya Julia
Victoria Garvey
Shrisha Shenoy
Llinos Davis
Annette Bolger
Timothy Hughes
Evita Pappa
Sian Saha
Rob Wilshire
Laura Tompsett
Nick Ridler
Luc Bugeja
Charlotte Humphrey
Carrie Colvin
Elizabeth Hood
Kim Wright
Dawn Davies
Amanda Cook
Lynda Connor
Marie Williams
Helen Goldring
Carl Murphy
Susan Anderson
Victoria Lacey
Atideb Mitra
Teresa Behan
Rachael Stead
Maria Newton
Elaine Heeney
Paul Hawkin
Julie Le Bas
Deborah Power
Andrew McGrath
Sarah Watson
Charlotte Hall
David Reicher
India Esam
Sumayer Sangera
Angeline Mbuyisa
Helen Newell
Faith Kibutu
Ramana Govindaraju
Susan Kilroy
Rachel Walker

Cheryl Graham
Sam Warnakulasuriya
Emilie Hoogenboom
Kayleigh Collins
Jen Mellersh
Jayne Evitts
Rebecca Purnell
Michael Swart
Jonathan Clouston
Jan Palmer
David Timbrell
Tracy Foster
Andrea Croucher
Baber Zaheer
Brendan Sloan
Alexandra Metcalfe
Martin Sylvester
Sanjeev Garg
Lisa-Jayne Cottam
Dorothy Hutchinson
Katrina Mellows
Manuela Brazil
Dilara Arslan
Emma Jackson
Concilia Dipura
Elizabeth Stones
Victoria Martinson
Amie Reddy
Senthil Nadarajan
Rebecca Francis
Stephanie Bell
Carol Buckman
Theresa Theobald
Nicola Wilkinson
Maria Fernandez
Filipa Santos
Fatima Seidu
Salma Begum
Viji Eldo
Nana Okine
David Wilcock
Caroline Tierney
Emma Robinson
Anthony Wilson
Cat Bounds
Ida Forro

Bret Claxton
Shereen Bano
Nicola Bosley
David Freeman
Karen Austin
Ellie Higgs
Jess Thrush
Al Hughes
Sue Smollen
Maria Scoble
Leanne Rees
Laura Jones
Karen Rahilly
S. Ramani Moonesinghe
Giuseppe Aresu
Sam Bampoe
Rachel Baumber
James Bedford
Matthew Bedford
Anne-Marie Bougeard,
Alexandra Brent
Martin Cripps
Jenny Dorey
Sharon Drake
Helen Ellicott
David Gilhooly
James Goodwin
Aleksandra Ignack,
Sandy Jackson
Irene Leeman
Jose Lourtie,
Peter Martin
Dorian Martinez
Dermot McGuckin
Dominic Olive
Arun Sahni,
Katie Samuel
Cristel Santos
Pritam Singh
Mike Swart
Christine Taylor
Olga Tucker
Abigail Vallance,
Ravinder Vohra
Duncan Wagstaff
Samantha Warnakulasuriya

Karen Williams
Jonathan Wilson
Kylie-Ellen Edwards
Georgina Singleton
Cecilia Vindrola-Padros
Jonathan Wilson
Martha Belete
Eleanor Warwick
Ravi Vohra
Alexandra Brent
Maria Chazapis
Vihara Dissanayake
Naomi Fulop
Mike Grocott
Helena Smith
